# Exemplar Learning and Memory Retrieval-Based Particle Swarm Optimization Algorithm with Engineering Applications

**DOI:** 10.3390/biomimetics10100708

**Published:** 2025-10-19

**Authors:** Shuying Zhang, Xiaohong Hu, Yue Gao, Minghan Gao, Yufei Zhang

**Affiliations:** 1 College of Computer Science and Technology, Beihua University, Jilin City 132013, China; jlzhangsy@beihua.edu.cn (S.Z.);; 2 College of Information Science and Technology, Jinan University, Guangzhou 510632, China; 3 College of Computer Science and Technology, Changchun University, Changchun 130022, China

**Keywords:** swarm intelligence, particle swarm optimization, engineering optimization

## Abstract

Particle swarm optimization (PSO) is a bio-inspired stochastic optimization algorithm that simulates the foraging behavior of birds. Despite its simplicity and efficiency, PSO often suffers from premature convergence and a poor balance between exploration and exploitation. These drawbacks mainly arise from its limited learning sources and rigid position update scheme. To address these issues, this paper proposes an enhanced PSO framework, termed Exemplar Learning and Memory Retrieval-Based Particle Swarm Optimization (EMPSO). The design of EMPSO is inspired by the learning, memory, and adaptation mechanisms observed in biological collectives. It integrates three complementary strategies to improve swarm intelligence. First, an elite exemplar learning mechanism aggregates the positional information of top-performing particles to construct a more reliable guidance vector. Second, a memory recall strategy retains exemplars that have recently contributed to global improvements and reuses them probabilistically with a recency bias, thus enabling effective knowledge inheritance. Third, an adaptive position update scheme assigns exploration- or exploitation-oriented behaviors to particles based on fitness ranking, promoting dynamic role differentiation within the swarm. Comprehensive experiments on the CEC2017 and CEC2022 benchmark suites demonstrate that EMPSO consistently outperforms six representative algorithms. Furthermore, applications to three engineering design problems and the optimal PMU placement task verify its robustness and practical effectiveness.

## 1. Introduction

Swarm intelligence (SI) algorithms originate from bio-inspired studies of collective behaviors observed in nature [1,2,3]. By mimicking the cooperation, competition, and information-sharing mechanisms exhibited by biological groups in decentralized and self-organized environments, SI algorithms provide adaptive and robust frameworks for solving complex optimization problems. Over the past few decades, numerous SI algorithms have been developed by emulating various biological phenomena, including Particle Swarm Optimization (PSO) [4], Ant Colony Optimization (ACO) [5], Whale Optimization Algorithm (WOA) [6], and White Shark Optimizer (WSO) [7], etc.

In contrast to gradient-based approaches that typically explore the neighborhood of local optima [8], heuristic algorithms are capable of conducting a more global search across the decision space [8,9]. Owing to their conceptual simplicity, scalability, and inherent robustness [10], SI algorithms have been widely applied in engineering design [11,12,13], machine learning [14,15,16], robotics [17,18,19], and industrial optimization [20,21,22], becoming an indispensable component of modern computational intelligence.

Among various SI paradigms, PSO stands as one of the most representative and influential bio-inspired algorithms [23]. Originating from the simulation of bird flocking and fish schooling behaviors, PSO models each candidate solution as a particle that iteratively adjusts its velocity and position through self-experience learning and social collaboration learning. These mechanisms enable particles to collectively approach promising regions in the search space. Due to its ease of implementation and strong capability of rapidly converging to high-quality solutions, PSO has been successfully applied to a wide spectrum of scientific and engineering optimization problems [24,25,26].

Nevertheless, the canonical PSO exhibits inherent shortcomings. It often suffers from premature convergence, losing population diversity too early and becoming trapped in local optima [27]. Furthermore, PSO struggles to maintain an appropriate balance between exploration and exploitation [28], particularly in deceptive or high-dimensional landscapes where both sustained diversity and fine-grained exploitation are essential.

To address these shortcomings, a large body of research has focused on improving PSO. Proposed strategies include adaptive parameter control [29,30] (e.g., inertia weight adjustment), hybridization with other metaheuristics [31,32], and alternative swarm topologies for information dissemination [33,34]. While such variants have achieved notable improvements, they remain limited by rigid update mechanisms, inefficient knowledge reuse, and inadequate responsiveness to the evolving fitness landscape. Specifically, traditional PSO relies heavily on current personal and global best positions as its sole learning sources, without mechanisms to preserve and exploit historical improvement trajectories or to adaptively differentiate learning strategies according to particle performance. As a result, the algorithm often exhibits unstable search dynamics and struggles to sustain an effective exploration–exploitation balance across different stages of optimization.

To address these limitations, this paper introduces a new PSO variant inspired by biological cognition and behavioral adaptation, termed Exemplar Learning and Memory Retrieval-Based Particle Swarm Optimization (EMPSO). The algorithm draws inspiration from the way individuals in biological collectives continuously optimize their behavior through experience accumulation, memory retrieval, and behavioral adjustment. EMPSO incorporates these cognitive elements into a unified framework consisting of three synergistic mechanisms: Elite Exemplar Learning (EEL), Superior Memory Recall (SMR), and Adaptive Position Update (APU).

Unlike conventional methods that rely solely on current best experiences, EMPSO explicitly models and reuses historical knowledge gained from successful improvements, providing a more stable and representative guidance direction for swarm evolution. The integration of EEL, SMR, and APU generates complementary search dynamics: EEL enhances convergence reliability by aggregating elite exemplars; SMR preserves valuable convergence trajectories and probabilistically reintroduces them to stimulate novel solution discovery; and APU adaptively allocates exploration or exploitation behaviors based on particle fitness ranking, enabling dynamic role differentiation within the swarm. Together, these mechanisms improve swarm adaptability across different optimization phases, enhance population diversity, and effectively alleviate premature stagnation.

The main contributions of this work can be summarized as follows:

- EEL: A mechanism that constructs a representative guidance vector by aggregating the positions of top-performing particles in a fitness-proportional manner, thereby improving swarm stability and convergence robustness.
- SMR: A dynamic memory bank that archives recent elite exemplars responsible for global improvements and reintroduces them with recency bias, enabling the algorithm to retain valuable knowledge and inspire exploration in promising directions.
- APU: A fitness-aware learning strategy that dynamically allocates exploitation-oriented or exploration-oriented updates according to relative particle fitness, yielding self-organized search dynamics that adapt to the current stage of optimization.

The remainder of this paper is organized as follows: Section 2 reviews related work on PSO and its advanced variants. Section 3 details the proposed EMPSO framework. Section 4 presents the experimental design and compares EMPSO with six representative SI algorithms on the CEC2017 and CEC2022 benchmark suites. Section 5 evaluates EMPSO on three real-world engineering design problems and the optimal PMU placement problem. Finally, Section 6 concludes the paper and outlines future research directions.

## 2. Preliminaries and Related Work

### 2.1. Particle Swarm Optimization

PSO is a population-based stochastic optimization algorithm originally proposed by Kennedy and Eberhart in 1995 [4], inspired by the social foraging and information-sharing behaviors observed in bird flocks and fish schools. Owing to its simplicity, low computational cost, and ability to efficiently exploit collective intelligence, PSO has become one of the most widely studied and applied metaheuristics in computational intelligence. Over the past decades, it has been successfully extended and tailored to solve a broad spectrum of optimization problems, ranging from continuous and combinatorial optimization to constrained, dynamic, and multi-objective scenarios.

In PSO, a population of candidate solutions, termed *particles*, jointly explores the search space while exchanging information to guide the search process. Each particle *i* at iteration *t* is represented by a position vector $x_{i}^{t}$ and a velocity vector $v_{i}^{t}$, which, respectively, denote a candidate solution and its search direction. Formally, for a *D*-dimensional problem with *N* particles,

$$x_{i}^{t}=\left(x_{i,1}^{t},x_{i,2}^{t},\ldots ,x_{i,D}^{t}\right),\;v_{i}^{t}=\left(v_{i,1}^{t},v_{i,2}^{t},\ldots ,v_{i,D}^{t}\right).$$

The search dynamics of PSO rely on two knowledge sources: the best position discovered by particle *i* itself (personal best, pbest), and the best position discovered by the entire swarm (global best, gbest). The velocity and position updates are as follows:

$$v_{i}^{t+1}=\omega v_{i}^{t}+c_{1}r_{1}\left({\mathrm{pbest}}_{i}-x_{i}^{t}\right)+c_{2}r_{2}\left(\mathrm{gbest}-x_{i}^{t}\right),$$

$$x_{i}^{t+1}=x_{i}^{t}+v_{i}^{t+1},$$

where ω is the inertia weight that balances global exploration and local exploitation, $c_{1}$ and $c_{2}$ are cognitive and social acceleration coefficients controlling the relative influence of self-experience and social learning, and $r_{1},r_{2}∼U\left(0,1\right)$ are stochastic factors that introduce randomness into the search process. To prevent excessive exploration in later iterations, ω is often linearly decreased with the iteration index *t*,

$$\omega =\omega_{\max}-\frac{\omega_{\max}-\omega_{\min}}{t_{\max}}\cdot t,$$

where $\omega_{\max}$ and $\omega_{\min}$ denote the upper and lower bounds of the inertia weight, respectively. Variants of PSO further refine this mechanism by adopting nonlinear decay, time-varying acceleration coefficients, or adaptive control rules to enhance search efficiency.

Although PSO has achieved remarkable success in diverse fields such as engineering design, feature selection, scheduling, and multi-objective optimization, it still suffers from several intrinsic weaknesses. The canonical PSO utilizes only two learning sources—the personal best and global best—lacking mechanisms for higher-order memory, structured knowledge sharing, or collaborative learning among subgroups. As a result, swarm diversity often decreases rapidly, causing premature convergence and stagnation in local optima, particularly in high-dimensional or multimodal problems. Moreover, PSO struggles to maintain a stable exploration–exploitation balance, frequently oscillating between excessive exploration of unpromising regions and over-exploitation near local attractors. These limitations have motivated extensive research into enhanced PSO variants, which aim to achieve more reliable and scalable performance across complex optimization scenarios.

### 2.2. Existing Improvements of PSO

Over the past two decades, a wide spectrum of PSO variants has been proposed to mitigate the aforementioned limitations. These efforts can be broadly categorized into three directions: (i) methods with improved topology structures, (ii) methods with dynamic parameter adjustment, and (iii) methods combining different optimization techniques. In the following subsections, we provide a systematic review of representative approaches in each category.

#### 2.2.1. Methods with Improved Topology Structures

Topology-improved PSO methods aim to strengthen population diversity and information propagation by redesigning the communication structure among particles. Li et al. [33] introduced the pyramid PSO, where particles are organized into a hierarchical pyramid according to their fitness levels. Particles within the same layer determine superiority through pairwise comparison: inferior particles collaborate with local winners, while superior particles interact with upper-layer elites. This hierarchical design significantly enhances population diversity and improves convergence behavior. Building upon this work, Jin et al. [35] further proposed an adaptive constraint-handling strategy based on the pyramid structure, which enhances the exploration capability of the swarm. Hu et al. [36] developed a centroid-based PSO, where a population centroid is generated at each iteration and used to replace the global best solution as the guidance source, thereby providing richer knowledge for position updates. Radwan et al. [37] proposed a three-stage framework, where the problem is decomposed into subproblems, solved via a cooperative multi-swarm approach, and complemented with a reaction mechanism to mitigate the diversity loss introduced by decomposition. Zhou et al. [38] developed a sub-swarm region-based solution selection mechanism to maintain diversity. By defining two neighborhood radii around global and local optima discovered during evolution, the swarm is encouraged to distribute more uniformly across the search space. Hong et al. [39] proposed an ensemble PSO framework that integrates adaptive covariance matrix learning, inertia-weighted PSO, and a sample-pool replacement mechanism, which collectively enhance convergence efficiency and robustness.

#### 2.2.2. Methods with Dynamic Parameter Adjustment

Dynamic parameter adaptation methods seek to balance exploration and exploitation by adjusting algorithmic parameters according to evolutionary progress or particle behavior. Liu et al. [29] proposed a weighting strategy based on the Sigmoid function, which takes into account both the distance from a particle to the global best position and the distance from the particle to its personal best position. This strategy enables adaptive adjustment of acceleration coefficients, thereby enhancing the convergence speed. Minh et al. [40] proposed the variable velocity strategy PSO, which introduces a new velocity term controlled by a linearly decreasing function, enabling more flexible position updates. Song et al. [41] introduced a fractional-order adaptive velocity parameter into PSO, which perturbs the swarm based on evolutionary states to improve the ability to escape local optima and explore the search space more thoroughly. Moazen et al. [42] proposed PSO-ELPM, where a cube-root inverse operation is employed to ensure smooth weight distribution, combined with an exponential mutation operator that adaptively adjusts mutation probability based on current and historical swarm states, thereby achieving a better balance between exploration and exploitation. Similarly, Meng et al. [43] proposed a novel PSO variant with an adaptive regulation of paradigm proportions and contraction coefficients during iterations. Moreover, a full-information search mechanism based on generational best solutions is introduced to help particles escape local optima and achieve improved global performance. Li et al. [44] proposed a novel variable weight coefficient based on evolutionary states to balance exploration and exploitation. Furthermore, multiple trial positions were used for each particle, and promising positions were selected by simultaneously leveraging the superiority and uncertainty of the ensemble. This approach ensures that the particle swarm maintains a large exploration space while controlling the convergence time.

#### 2.2.3. Methods Combining Different Optimization Techniques

Hybridization approaches aim to integrate complementary mechanisms from other optimization algorithms into PSO, thereby compensating for the inherent limitations of a single strategy. Li et al. [45] proposed the multi-component PSO algorithm, where four distinct PSO variants are incorporated into a strategy pool. A leader-learning mechanism is employed to facilitate knowledge sharing and guide global convergence, enabling the swarm to exploit the complementary advantages of different PSO paradigms in a cooperative manner. Şenel et al. [46] proposed a hybrid algorithm combining PSO and the Grey Wolf Optimizer (GWO), in which a fraction of PSO particles are probabilistically replaced by GWO-enhanced solutions. This hybridization effectively leverages the exploitation strength of PSO and the exploration ability of GWO. Other studies have integrated PSO with evolutionary operators. Liu et al. [47] proposed the integration of evolutionary game theory into the research of PSO algorithms, combining four classical variants of PSO algorithms with different exploration and exploitation capabilities. The population is divided into two subpopulations, with more advantageous strategies achieving a higher execution probability in the larger subpopulation. A hybrid ML–TSO approach [48] combined transient search optimization with learning-based modeling to minimize power generation costs in both classical and probabilistic optimal power flow problems. An ANN–PSO model [49] was embedded within a probabilistic machine learning framework to improve the prediction accuracy of soil desiccation cracking under environmental uncertainty. A PSO–ant lion optimization hybrid [50] was applied to optimize a probabilistic neural network for wind speed forecasting, achieving faster convergence and higher prediction accuracy than conventional models.

#### 2.2.4. Discussion

In summary, existing improvements of PSO have made remarkable progress in addressing premature convergence and enhancing the exploration–exploitation balance. Topology-based strategies mainly enrich the communication structure to preserve diversity, parameter adaptation methods enable responsive adjustments to evolutionary states, and hybridization approaches introduce external mechanisms to mitigate PSO’s inherent weaknesses. Probabilistic machine learning methods explicitly model uncertainty and inter-sample covariance to guide search decisions. However, most of these approaches tend to emphasize one aspect (e.g., diversity preservation or convergence acceleration) while lacking a unified design that systematically integrates multiple knowledge sources and adaptive mechanisms.

To this end, we argue that further progress requires a more holistic framework that simultaneously leverages elite information, historical knowledge, and adaptive search dynamics. Motivated by this perspective, our proposed algorithm introduces three synergistic mechanisms: (1) EEL, which aggregates knowledge from multiple high-quality particles to prevent over-reliance on a single leader; (2) SMR, which reuses superior historical exemplars to reintroduce valuable search trajectories when stagnation occurs; and (3) APU, which allocates distinct update rules to different subgroups of particles according to their fitness levels. Together, these mechanisms provide complementary strengths, offering a more stable balance between exploration and exploitation across diverse problem scenarios.

## 3. Proposed Algorithm

**Figure 1:**
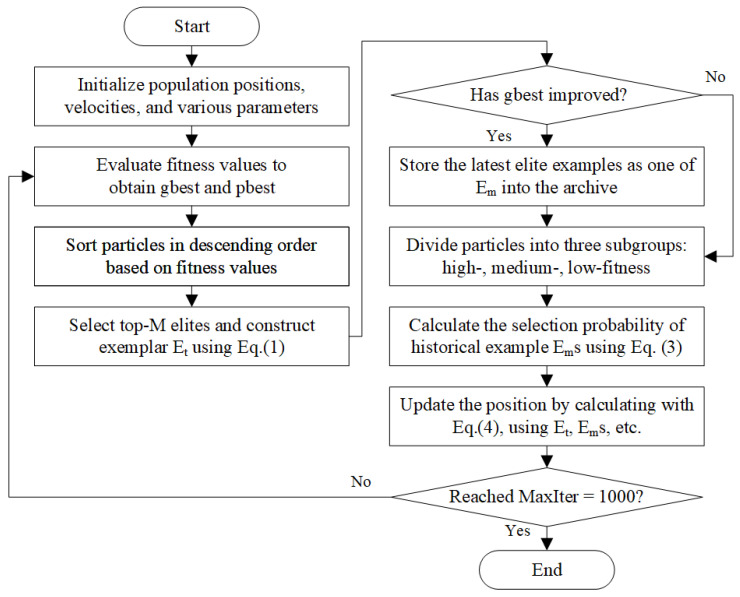
Overall flowchart of EMPSO.

In this section, we present the proposed variant of PSO, named EMPSO, which integrates three synergistic mechanisms: EEL, SMR, and APU. These mechanisms are designed to enrich the knowledge sources available to the swarm, enhance the utilization of valuable information, and dynamically balance exploration and exploitation during the search process. The overall flowchart of EMPSO is shown in Figure 1.

### 3.1. Elite Exemplar Learning (EEL)

In the canonical PSO, each particle updates its position primarily based on its personal best solution $pbest_{i}$ and the global best solution gbest. Such a limited knowledge source often causes particles to be overly attracted to the global optimum, leading to rapid aggregation around a single region of the search space. Consequently, diversity is reduced, which may result in premature convergence and suboptimal performance.

To address this issue, we propose an *EEL* mechanism that exploits multiple elite particles to generate a knowledge exemplar suitable for minimization tasks. Specifically, at iteration *t*, all particles are ranked in ascending order according to their objective values, since a smaller value indicates better fitness. Let $E_{t}$ denote the set of the top-*M* elite particles selected from the population $P_{t}$, where M=0.3N. Each elite particle $x_{j}\in E_{t}$ is assigned a weight inversely proportional to its objective value, and the elite exemplar $E_{t}$ is constructed as follows:

(1) $$E_{t}=\sum_{x_{j}\in E_{t}}w_{j}\cdot x_{j},\;w_{j}=\frac{1/f(x_{j})}{\sum_{x_{k}\in E_{t}}1/f\left(x_{k}\right)},$$

where $f(x_{j})$ denotes the objective (fitness) value of particle $x_{j}$, and a smaller $f(x_{j})$ corresponds to higher quality. By aggregating knowledge from multiple elites through inverse-value weighting, $E_{t}$ provides a more representative and balanced exemplar to guide the swarm toward regions with lower objective values, thereby enhancing search directionality and maintaining population diversity.

### 3.2. Superior Memory Recall (SMR)

Most existing PSO variants rely exclusively on the current pbest and gbest, while neglecting historical knowledge. This lack of memory may cause valuable optimization trajectories to be forgotten, limiting the algorithm’s ability to escape from stagnation.

To reinforce knowledge reusability, we introduce the *SMR* mechanism. At each iteration *t*, if the best solution $x_{t}^{*}$ obtained in the current population improves upon the historical global best gbest, the elite exemplar $E_{t}$ is considered *superior knowledge* and stored in a memory archive M. Formally,

(2) $$\mathrm{if}\;f\left(x_{t}^{*}\right)<f\left(gbest\right),\;M\leftarrow M\cup \left\{E_{t}\right\}.$$

When a particle’s search ability is limited (i.e., its fitness level does not reach the elite subset of the population), the exemplars $E^{m}\in M$ are probabilistically selected to guide its position update. The probability of selecting $E^{m}$ is defined as Equation (3):

(3) $$P\left(E^{m}\right)=\frac{\exp(-\lambda \left(t-t_{m}\right))}{\sum_{E^{k}\in M}\exp\left(-\lambda \left(t-t_{k}\right)\right)},$$

where $t_{m}$ denotes the iteration when $E^{m}$ was stored, and λ is a decay parameter controlling the preference for recent memory. In this way, more recent exemplars are more likely to be reused, enabling the swarm to reintroduce successful historical trajectories and escape local optima.

### 3.3. Adaptive Position Update (APU)

In standard PSO, all particles follow the same update strategy, which may lead to an imbalance between exploration and exploitation. To address this limitation, we propose an APU mechanism that dynamically assigns heterogeneous search behaviors to particles based on their relative fitness ranking.

At iteration *t*, the population $P_{t}$ is divided into the following three subgroups:

- High-fitness particles: focused on exploitation, emphasizing fine-tuning around the best-known regions.
- Medium-fitness particles: assigned a hybrid strategy, balancing exploration and exploitation.
- Low-fitness particles: dedicated to exploration, encouraging escape from inferior regions.

Formally, the velocity update rule for particle *i* is defined as Equation (4),

(4) $$v_{i}^{t+1}=\left\{\begin{array}{cc} \omega v_{i}^{t}+c_{1}r_{1}\left({\mathrm{pbest}}_{i}-x_{i}^{t}\right)+c_{2}r_{2}\left(E_{t}-x_{i}^{t}\right), & i\in \mathrm{high}\text{-}\mathrm{fitness}\;\mathrm{group},\\ \omega v_{i}^{t}+c_{1}r_{1}\left(E^{m1}-x_{i}^{t}\right)+c_{2}r_{2}\left(\mathrm{gbest}-x_{i}^{t}\right), & i\in \mathrm{medium}\text{-}\mathrm{fitness}\;\mathrm{group},\\ \omega v_{i}^{t}+c_{1}r_{1}\left(E^{m2}-x_{i}^{t}\right)+c_{2}r_{2}\left(E^{m3}-x_{i}^{t}\right), & i\in \mathrm{low}\text{-}\mathrm{fitness}\;\mathrm{group}, \end{array}\right.$$

where ω is the inertia weight, $c_{1}$ and $c_{2}$ are acceleration coefficients, and $r_{1},r_{2}∼U\left(0,1\right)$. Here, $E_{t}$ denotes the current elite exemplar, while $E^{m1}$, $E^{m2}$, and $E^{m3}$ represent three exemplars retrieved from memory. These examples are independently sampled with replacement according to the probabilities computed by Equation (3), so they may be the same or different across different selections. The pseudocode, for example, sampling is shown in Algorithm 1.
**Algorithm 1:** SampleExemplar_WithReplacement (M,t,λ)
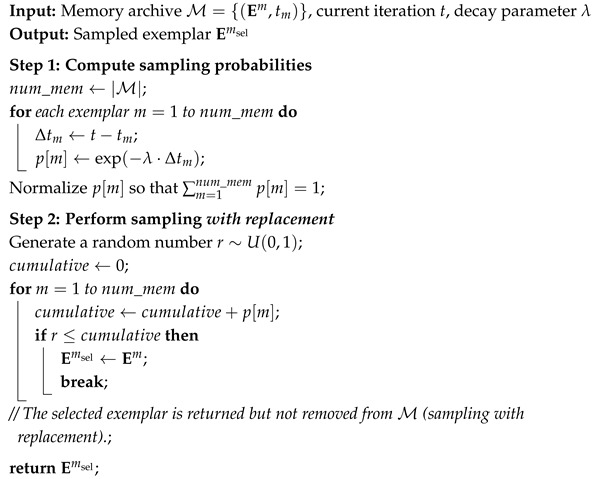


The adaptive mechanism assigns complementary search roles to different particle subgroups, maintaining a dynamic balance between exploration and exploitation. High-quality particles use the elite exemplar to reduce premature convergence, medium-quality particles exploit memorized exemplars to escape local optima, and low-quality particles follow multiple stored exemplars to quickly reach promising regions. This design improves search efficiency and accelerates convergence.

### 3.4. Pseudocode of the Proposed Algorithm

The overall procedure of the proposed PSO variant is summarized in Algorithm 2.
**Algorithm 2:** Proposed PSO with EEL, SMR, and APU
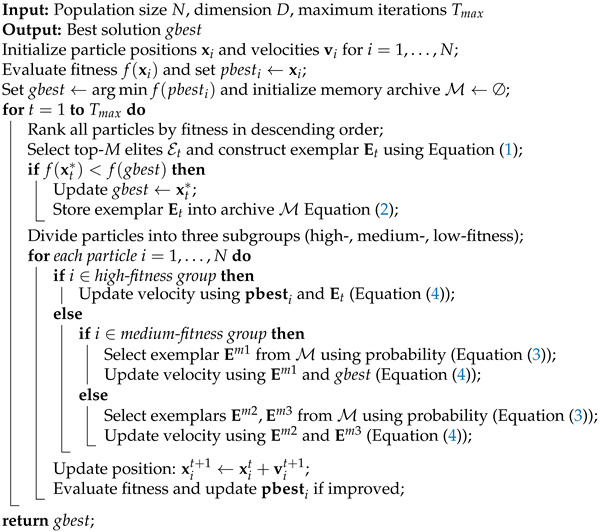


### 3.5. Complexity Analysis

The proposed algorithm introduces three additional mechanisms on top of the standard PSO framework. The computational cost can be analyzed as follows:

- EEL requires sorting the population by fitness in O(NlogN) time and aggregating the top-*M* elites. Since M≪N, the overall cost is dominated by sorting.
- SMR involves updating the memory archive and sampling exemplars, which incurs at most O(M) additional cost per iteration.
- APU modifies the velocity update rules without changing the complexity, i.e., O(ND), where *D* is the problem dimension.

Therefore, the overall computational complexity per iteration is

(5) O(ND+NlogN).

Since D≫logN in most real-world optimization tasks, the additional cost introduced by EEL and SMR is negligible compared to the standard PSO. Meanwhile, the proposed mechanisms significantly enhance the diversity and knowledge exploitation of the swarm, which improves the algorithm’s robustness and convergence behavior.

## 4. Experiments on the Benchmark Suite

This section presents a comprehensive empirical study to evaluate the performance of the proposed EMPSO algorithm. The experiments are organized into three groups. First, EMPSO is compared with six representative algorithms on the widely used CEC2017 benchmark suite [51] and the more recent CEC2022 benchmark suite [52]. Second, a sensitivity analysis is conducted to investigate the impact of the parameter on algorithmic performance. Finally, multiple ablation studies are performed to assess the effectiveness of the three position update strategies.

### 4.1. Experimental Setup

#### 4.1.1. Computing Platform

All experiments were executed on a workstation equipped with an Intel(R) Xeon(R) CPU E5-2696 v3 and 64 GB of RAM. The operating system was Windows 11 (version 24H2), and the implementation was carried out using MATLAB 2024a.

#### 4.1.2. Parameter Settings

To ensure a fair comparison, all competing algorithms were configured with standard parameter settings commonly adopted in the literature. Specifically, the maximum number of iterations was set to T=1000, the population size to N=100. The remaining algorithm-specific parameters are summarized in Table 1. Since population-based metaheuristics are inherently stochastic, each benchmark function was independently tested 30 times, and the mean and standard deviation of the obtained results were reported to mitigate the influence of randomness.

#### 4.1.3. Benchmark Suites

CEC2017 Benchmark Suite: The CEC2017 benchmark suite consists of 29 continuous optimization test functions designed to comprehensively evaluate algorithmic performance under varying levels of problem complexity. The suite includes unimodal functions (F1–F3), multimodal functions (F4–F10), hybrid functions (F11–F20), and composition functions (F21–F30), covering a broad spectrum of search landscape characteristics. Test dimensions are typically set to 30 or 50 to balance computational cost and discriminative power. All functions are shifted and rotated to introduce translation and rotation invariance, thereby making CEC2017 a widely adopted benchmark for assessing the robustness and generalization ability of optimization algorithms.

CEC2022 Benchmark Suite: The CEC2022 benchmark suite comprises 12 single-objective bound-constrained optimization problems that more closely emulate the complexity of real-world applications. Compared with CEC2017, this suite enhances scalability, landscape diversity, and inter-variable dependency, providing a more rigorous test of an algorithm’s performance under nonlinear and highly correlated conditions. It includes unimodal (F1), multimodal (F2–F5), hybrid (F6–F8), and composition functions (F9–F12), all subject to shifting, rotation, and dynamic scaling. These design features ensure that the CEC2022 suite serves as a challenging and realistic platform for benchmarking the stability, adaptability, and convergence efficiency of advanced optimization algorithms.

### 4.2. Comparative Results on the CEC2017 Benchmark Suite

**Figure 2:**
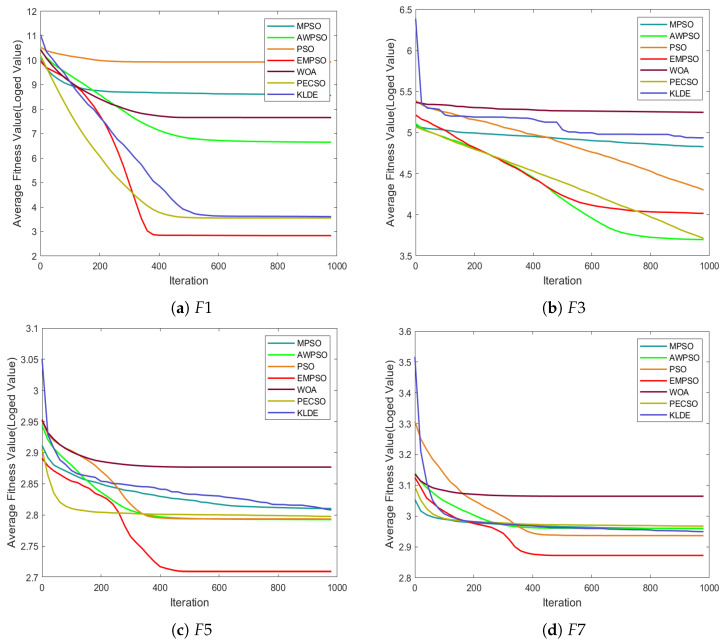

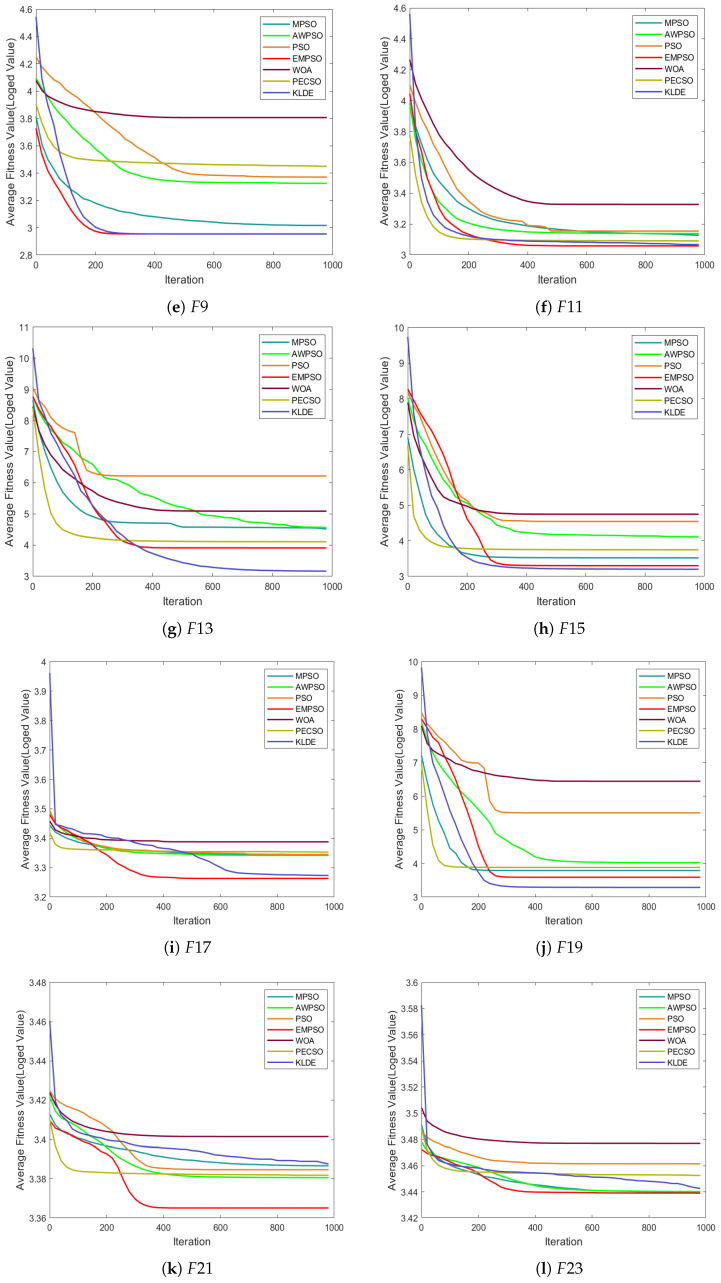

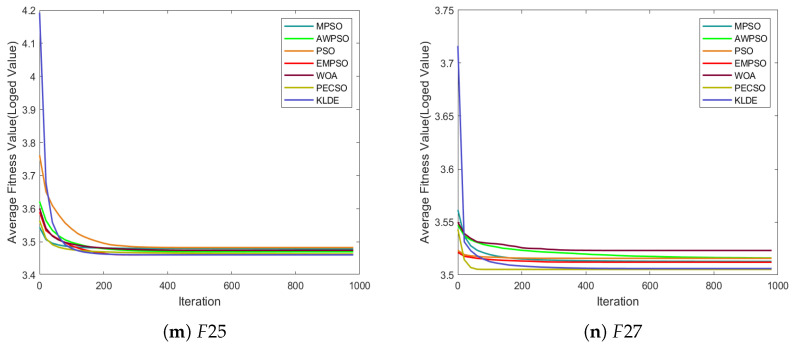
Average convergence trends of EMPSO and 6 comparison algorithms on selected CEC2017 functions.

To thoroughly assess the adaptability and competitiveness of EMPSO in solving complex optimization problems, a comprehensive set of experiments was conducted on the CEC2017 test suite. The numerical results, including mean errors, standard deviations, overall rankings, and total running time, are summarized in Table 2; the convergence trajectories on several representative functions are plotted in Figure 2; and the statistical significance tests together with the final ranking outcomes are reported in Table 3.

#### 4.2.1. Accuracy Comparison

Table 2 clearly demonstrates the superior accuracy of EMPSO across the CEC2017 benchmarks. EMPSO achieved the best average rank of 1.52, benefiting from obtaining the best fitness values on 17 out of the 29 functions, which is significantly better than all competing algorithms. Among the baselines, KLDE followed with average ranks of 2.48, while PSO and WOA exhibited overall weaker performance. Moreover, EMPSO not only yielded lower mean errors but also exhibited smaller variances, reflecting more stable convergence behavior.

Further analyses across different function categories reveal the consistent superiority of EMPSO. For unimodal functions (F1–F3), which mainly test exploitation capability, EMPSO achieves the best overall performance. This advantage is attributed to the effectiveness of the EEL strategy in conducting fine-grained local searches around the global optimum. For multimodal functions (F4–F10), where escaping from local optima is crucial, EMPSO again outperforms the other algorithms. The results indicate that the SMR mechanism enhances the swarm’s ability to traverse complex landscapes. For hybrid functions (F11–F20), which combine multimodality with separable structures, EMPSO attains the best performance on five test functions. This outcome validates that the APU strategy promotes a well-balanced search behavior between exploration and exploitation. Finally, for the most challenging composite functions (F21–F30) characterized by intricate multilayer structures, EMPSO maintains a clear advantage. It achieves the best results on seven functions. Even when not ranking first, EMPSO consistently remains among the top performers, demonstrating its strong adaptability and robustness across diverse problem landscapes.

#### 4.2.2. Convergence Trend Analysis

To further evaluate the search dynamics of EMPSO, we report the mean convergence curves over 30 independent runs on 14 representative CEC2017 benchmark functions, as shown in Figure 2. Overall, EMPSO demonstrates a clear advantage in convergence accuracy compared to the competing algorithms, achieving superior results on 9 out of the 14 selected functions. These results confirm the effectiveness and reliability of the proposed hierarchical strategy in guiding the search process.

A closer examination reveals that, on several functions such as F1, F5, F7, and F21, EMPSO exhibits an “S”-shaped convergence pattern. This behavior indicates that EMPSO is capable of maintaining steady progress in the early and middle phases while effectively escaping local optima in later stages. Such adaptability can be attributed to the exemplar-based learning mechanism, which leverages historical high-quality solutions to introduce promising knowledge into the swarm when the search stagnates. Consequently, EMPSO achieves more stable accuracy improvements, highlighting its ability to balance exploration and exploitation across diverse problem landscapes.

#### 4.2.3. Statistical Analysis via the Wilcoxon Rank-Sum Test

To further examine the statistical significance of the performance differences between EMPSO and the six representative algorithms, the well-known non-parametric Wilcoxon rank-sum test [55] was employed at a significance level of 0.05. The results are summarized in Table 3, where the symbols “−”, “≈”, and “+” denote that EMPSO performs significantly worse, statistically equivalent, or significantly better than the corresponding algorithm, respectively.

From Table 3, it can be observed that EMPSO exhibited statistically significant superiority on the majority of benchmark functions. In particular, EMPSO outperformed MPSO, WOA, and PSO on 29 out of 30 functions, with no instances of inferior performance, demonstrating strong robustness. Against AWPSO and PECSO, EMPSO also achieved 26 or 27 statistically significant wins, with only 2 losses in each case. KLDE’s performance is relatively close, but EMPSO still achieved 17 significant advantages. Overall, the Wilcoxon test further confirms the effectiveness of EMPSO and its clear statistical advantage over the compared algorithms.

### 4.3. Comparative Results on the CEC2022 Benchmark Suite

To further evaluate the adaptability and competitiveness of EMPSO, we conducted additional tests on 12 benchmark functions from the more recent CEC2022 test suite. The same six representative algorithms mentioned earlier were selected as baseline methods: KLDE, MPSO, AWPSO, PECSO, WOA, and classical PSO. The numerical experimental results, including average error, standard deviation, overall ranking, and runtime, are summarized in Table 4.

Table 4 reports the comparative results of EMPSO and six representative algorithms on twelve CEC2022 benchmark functions. Overall, EMPSO achieves the best mean rank of 2.08, outperforming all competitors, followed by AWPSO (3.08) and KLDE (3.25).

Specifically, EMPSO secures the top performance on five functions (F1, F4, F5, F7, and F9) and maintains competitive stability across the rest. Its advantages are particularly pronounced on unimodal and hybrid composition functions (e.g., F1–F5), where accurate exploitation and exemplar-guided learning contribute to consistent convergence. Although KLDE slightly surpasses EMPSO on certain multimodal cases (e.g., F6 and F3), EMPSO still demonstrates robust overall adaptability. Moreover, EMPSO exhibits a favorable computational efficiency, with an average runtime of 564.84 s, significantly lower than KLDE (12,464.14 s) while remaining comparable to lightweight variants such as AWPSO and MPSO. These results substantiate the effectiveness and generalizability of the proposed learning and memory retrieval mechanisms under diverse optimization landscapes.

### 4.4. Parameter Sensitivity Analysis

To further investigate the sensitivity of EMPSO to its control parameter λ, we conducted additional experiments on the CEC2017 benchmark set, where λ was configured as {0,0.1,0.2,0.3,0.5}. The averaged performance over all 30 functions is summarized in Table 5. The parameter λ regulates the preference of EMPSO when recalling historical exemplars. Specifically, λ=0 implies that all recorded exemplars are selected with nearly uniform probability, while λ=0.5 indicates a strong bias towards the most recent exemplars. Thus, λ can be interpreted as a memory decay factor, controlling the balance between short-term and long-term experience in guiding the swarm dynamics.

As shown in Table 5, the choice of λ demonstrates considerable robustness. Across all tested configurations, EMPSO consistently outperforms its competitors, as reported in Table 2, confirming that λ plays a non-trivial role in shaping the search behavior of the algorithm. Different settings of λ result in distinct search dynamics. For instance, on certain functions such as F5, F8, F24, and F26, the performance deteriorates as λ increases, which may be attributed to excessive reliance on short-term memory that restricts global exploration. Conversely, on other functions such as F9, F28, and F30, larger values of λ lead to improved performance, suggesting that emphasizing recent exemplars can accelerate convergence when the fitness landscape exhibits relatively stable local structures. These observations indicate that extreme values of λ may enhance EMPSO’s adaptability for particular problem classes, but they also introduce higher variance in performance across tasks.

Nevertheless, a moderate choice of λ appears to achieve the best trade-off, yielding the best results on 16 out of the 29 benchmark functions. This also highlights the effectiveness of the SMR strategy, which provides a balanced utilization of both recent and distant memory, thereby reducing the risk of premature convergence while maintaining sufficient exploitation ability. Based on these results and to ensure fairness in comparisons, λ=0.2 is adopted as the default configuration in all experiments.

### 4.5. Ablation Study

To further investigate the contribution of different components in EMPSO, we conducted an ablation study on several representative CEC2017 functions. Specifically, three degraded variants were implemented by removing the high-level, middle-level, and low-level strategies, respectively. The detailed results are summarized in Table 6.

Overall, EMPSO achieves the best mean rank (1.13) across all test functions, significantly outperforming its variants and the canonical PSO. This demonstrates that the hierarchical strategy design of EMPSO is essential for balancing exploration and exploitation.

When the high-level strategy is removed (w/o_High), the performance deteriorates markedly on most functions, such as F1, F3, F13, F15, and F19, where the mean errors increase by several orders of magnitude compared to EMPSO. This degradation highlights the critical role of the high-level mechanism in promoting global guidance and preventing premature convergence.

The removal of the middle-level strategy (w/o_Middle) leads to moderately reduced performance; although this variant performs better than w/o_High, it remains consistently inferior to EMPSO, particularly on F3, F7, and F11, indicating that the middle-level strategy is vital for sustaining population diversity and enhancing robustness.

In contrast, the variant without the low-level strategy (w/o_Low) exhibits competitive or even superior results on certain problems, such as F5, suggesting that the low-level component primarily contributes to fine-grained exploitation and local refinement, whose benefits may be problem-dependent. Nonetheless, when considering the overall performance across all test functions, EMPSO still surpasses all its variants, while the canonical PSO shows the poorest results (mean rank = 5.00).

These findings confirm that each hierarchical level provides complementary benefits, and their synergistic integration enables EMPSO to achieve superior accuracy, stability, and adaptability across diverse optimization landscapes.

## 5. Simulation on Engineering Optimization Problems

To further evaluate the practical effectiveness of the proposed algorithm, we conducted simulations on three widely used constrained engineering design problems: the three-bar truss design [56], the pressure vessel design [57], and the tension/compression spring design [58]. These problems are representative of real-world engineering optimization tasks, which are typically characterized by nonlinearity, discrete variables, and complex constraints. The detailed formulations are presented below.

### 5.1. Problem Formulations

#### 5.1.1. Three-Bar Truss Design

**Figure 3:**
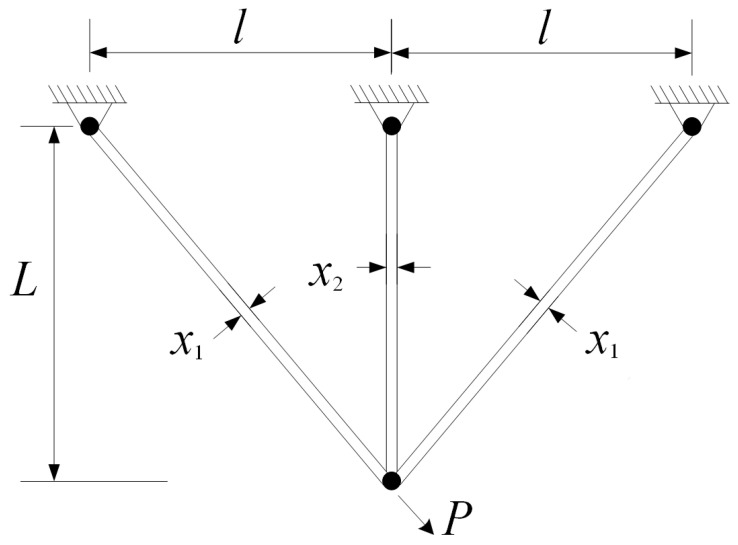
Schematic of the three-bar truss structure.

The three-bar truss design is a classical benchmark in structural optimization. The objective is to minimize the overall weight of a planar truss while ensuring that the stress on each bar and the displacement at the loaded joint remain within acceptable limits. This problem is widely adopted to test the capability of optimization algorithms in handling nonlinear stress–displacement interactions. The structure of the three-bar truss is illustrated in Figure 3.

**Expression:**

$$f\left(x\right)=\left(2\sqrt{2}x_{1}+x_{2}\right)\cdot l\cdot \rho ,$$

where $x_{1}$ and $x_{2}$ denote the cross-sectional areas of truss members, *l* is the member length, and ρ is the material density.

**Constraints:**

$$\begin{array}{ccc} g_{1}\left(x\right) & \leq 0\; & (\mathrm{stress}\;\mathrm{in}\;\mathrm{bar}\;1),\\ g_{2}\left(x\right) & \leq 0\; & (\mathrm{stress}\;\mathrm{in}\;\mathrm{bar}\;2),\\ g_{3}\left(x\right) & \leq 0\; & (\mathrm{stress}\;\mathrm{in}\;\mathrm{bar}\;3),\\ g_{4}\left(x\right) & \leq 0\; & (\mathrm{displacement}\;\mathrm{limit}). \end{array}$$

**Variable Scope:**

$$0\leq x_{1},x_{2}\leq 1.0\;\;\left({\mathrm{in}}^{2}\right).$$

#### 5.1.2. Pressure Vessel Design

**Figure 4:**
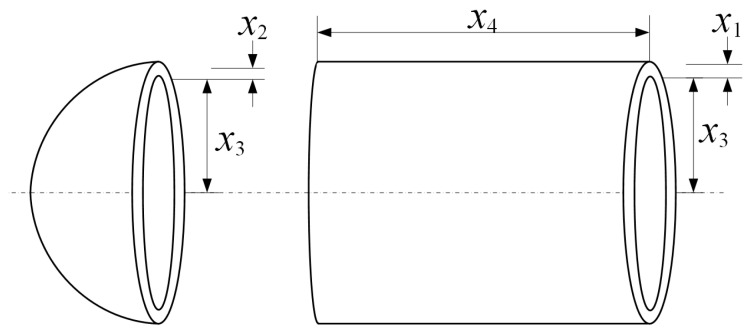
Schematic of the pressure vessel design problem.

The pressure vessel design problem is a well-known engineering benchmark involving both continuous and discrete decision variables. The goal is to minimize the total cost of material, forming, and welding, subject to safety and design requirements. Due to the mixed-variable nature and nonlinear constraints, this problem is particularly challenging for evolutionary algorithms. The schematic representation of the vessel structure is shown in Figure 4.

**Expression:**

$$f\left(x\right)=0.6224x_{1}x_{3}x_{4}+1.7781x_{2}x_{3}^{2}+3.1661x_{1}^{2}x_{4}+19.84x_{1}^{2}x_{3},$$

where $x_{1}$ and $x_{2}$ are the thickness of the shell and head, $x_{3}$ is the inner radius, and $x_{4}$ is the length of the cylindrical section.

**Constraints:**

$$\begin{array}{cc} g_{1}\left(x\right) & =-x_{1}+0.0193x_{3}\leq 0,\\ g_{2}\left(x\right) & =-x_{2}+0.00954x_{3}\leq 0,\\ g_{3}\left(x\right) & =-\pi x_{3}^{2}x_{4}-\frac{4}{3}\pi x_{3}^{3}+1,296,000\leq 0,\\ g_{4}\left(x\right) & =x_{4}-240\leq 0. \end{array}$$

**Variable Scope:**

$$\begin{array}{c} x_{1}\in \{1,2,\ldots ,99\}\times 0.0625,\\ x_{2}\in \{1,2,\ldots ,99\}\times 0.0625,\\ 10\leq x_{3}\leq 200,\\ 10\leq x_{4}\leq 200. \end{array}$$

#### 5.1.3. Tension/Compression Spring Design

**Figure 5:**
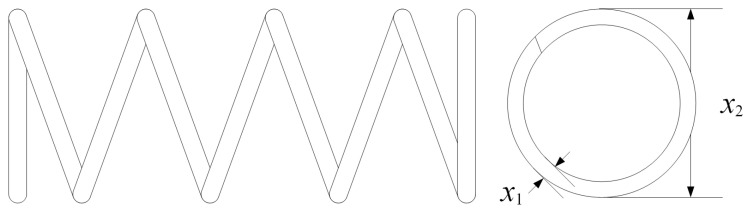
Schematic of the tension/compression spring design problem.

The tension/compression spring design problem focuses on minimizing the spring’s weight while ensuring that it meets the requirements on shear stress, deflection, and frequency. This benchmark reflects practical challenges in mechanical design, as it involves highly nonlinear constraints and conflicting objectives. The schematic diagram of the spring structure is depicted in Figure 5.

**Expression:**

$$f\left(x\right)=\frac{\pi^{2}x_{2}x_{3}x_{1}^{2}}{4},$$

where $x_{1}$ is the wire diameter, $x_{2}$ is the mean coil diameter, and $x_{3}$ is the number of active coils.

**Constraints:**

$$\begin{array}{ccc} g_{1}\left(x\right) & =\frac{8F_{\max}x_{2}}{\pi x_{1}^{3}}-S\leq 0\; & (\mathrm{shear}\;\mathrm{stress}),\\ g_{2}\left(x\right) & =l_{\max}-\left(\frac{F_{\max}}{K}+1.05\left(x_{3}+2\right)x_{1}\right)\geq 0\; & \left(\mathrm{deflection}\right),\\ g_{3}\left(x\right) & =\frac{x_{2}}{x_{1}}-3\geq 0,\\ g_{4}\left(x\right) & =15-\frac{x_{2}}{x_{1}}\geq 0. \end{array}$$

**Variable Scope:**

$$0.05\leq x_{1}\leq 2.0,\;0.25\leq x_{2}\leq 1.3,\;2\leq x_{3}\leq 15.$$

### 5.2. Experimental Setup

For each engineering optimization problem, the proposed EMPSO was compared against five representative SI algorithms, namely AWPSO, PECSO, WOA, WSO, and the canonical PSO. All algorithms were executed under identical termination conditions, with the maximum number of function evaluations (FEs) set to $1.0\times 10^{5}$. Each algorithm was independently run 30 times to ensure statistical reliability.

### 5.3. Results and Discussion

For the three-bar truss design problem (Table 7), all algorithms were able to converge to the vicinity of the known optimum (263.89584). Nevertheless, EMPSO stands out in terms of stability, achieving an almost negligible variance ($9\times 10^{-6}$). Compared with advanced variants such as AWPSO and PECSO, EMPSO yields lower variability, indicating that the exemplar-driven search mechanism effectively preserves convergence reliability in low-dimensional structural design tasks.

In the pressure vessel design problem (Table 8), the performance differences among algorithms become more pronounced. EMPSO consistently identifies solutions close to $6.06\times 10^{3}$, with the best solution of 6059.71, which is highly competitive with the best-known designs reported in the literature. Its average performance (6073.07) is significantly superior to AWPSO (7826.72) and WOA (6728.76), both of which exhibit larger variances. The narrow spread of EMPSO’s results (Std. =15.53) highlights its efficiency and robustness when handling mixed-integer constraints. These findings emphasize the advantage of exemplar-based learning in guiding the population toward feasible and high-quality regions within complex design landscapes.

For the tension/compression spring design problem (Table 9), EMPSO again demonstrates competitive performance, achieving the best solution of 0.01267, an average value of 0.01271, and a very small variance. Although classical PSO also attains near-optimal solutions with slightly smaller variance, both methods reliably converge to the global optimum in this relatively smooth search space. By contrast, AWPSO and PECSO exhibit inferior mean performance, confirming that EMPSO maintains robustness even on less challenging problems.

Overall, across the three constrained engineering benchmarks, EMPSO consistently achieves highly competitive or superior results in terms of best and mean objective values while maintaining low variance. Its advantage is particularly evident in the pressure vessel problem, where the coexistence of discrete and continuous variables poses a considerable challenge for standard metaheuristics. These results demonstrate that EMPSO provides a robust and flexible approach for addressing practical engineering design tasks characterized by complex constraints and heterogeneous decision variables.

## 6. Simulation on Optimal PMU Placement Problem

### 6.1. Problem Formulation

The Optimal PMU Placement (OPP) [8,9,59] problem aims to determine the minimum number of Phasor Measurement Units (PMUs) and their optimal locations in a power system to ensure full network observability.

Consider a power system with *N* buses and *L* transmission lines. Let

$$x_{i}=\left\{\begin{array}{cc} 1, & \mathrm{if}\;a\;\mathrm{PMU}\;\mathrm{is}\;\mathrm{installed}\;\mathrm{at}\;\mathrm{bus}\;i,\\ 0, & \mathrm{otherwise}. \end{array}\right.\;i=1,2,\ldots ,N$$

denote the binary decision variable representing PMU placement.

The OPP problem can be formulated as the following optimization problem:

(6) $$\begin{array}{cc} \min & \sum_{i=1}^{N}x_{i}\\ s.t. & \mathrm{Each}\;\mathrm{bus}\;\mathrm{is}\;\mathrm{observable}\;\mathrm{either}\;\mathrm{directly}\;\mathrm{or}\;\mathrm{via}\;a\;\mathrm{connected}\;\mathrm{PMU}\\ \; & \mathrm{Observability}\;\mathrm{constraints}\;\mathrm{include}\;\mathrm{zero}-\mathrm{injection}\;\mathrm{buses}\;\mathrm{where}\;\mathrm{applicable}\\ \; & x_{i}\in \left\{0,1\right\},\;i=1,2,\ldots ,N \end{array}$$

where the objective function (Equation (6)) minimizes the total number of PMUs. A bus is considered observable if either:

1. A PMU is installed at the bus itself, or
2. The bus is connected to another bus equipped with a PMU.

For zero-injection buses (buses with no load generation), additional observability rules are applied: if all neighboring buses except one are observable, the zero-injection bus can help infer the voltage of the remaining unobserved bus. This reduces the required number of PMUs compared to a naive placement.

Even though representative optimization methods (such as [8,9]) have already satisfactorily addressed this PMU placement problem, the issue still holds research value and can effectively characterize the practical application capability and robustness of a new method.

### 6.2. Experimental Setup

To validate the effectiveness of the proposed EMPSO algorithm for the OPP problem, extensive experiments were conducted on the IEEE 30-bus, IEEE 39-bus, IEEE 57-bus, and IEEE 118-bus test systems.

EMPSO is compared with the binary particle swarm optimization (BPSO) [60] algorithm and the binary bat algorithm (BBA) [61]. For each algorithm, the performance is evaluated through four key indicators reported over 30 independent runs: the average number of PMUs obtained, the standard deviation of the number of PMUs obtained, the minimum number of PMUs achieved in the optimal case, and the maximum number of PMUs in the sub-optimal case. Additionally, the PMU placement configuration from the best run is documented for each test system to provide insight into the optimal solutions achieved. During testing, we set the population size to 100 and the maximum number of iterations to 1000.

It should be noted that EMPSO was originally designed for continuous optimization problems. Therefore, when applying it to binary optimization tasks, several minor adjustments were introduced without altering the main algorithmic framework. Specifically, each particle’s position was interpreted as a continuous probability within the range [0,1], and subsequently thresholded to a binary selection vector before being evaluated by the functions—following the common practice in binary PSO adaptations. In addition, the position update boundaries were constrained to [0,1], and the final evaluation employed a binarization step to ensure valid discrete representations.

### 6.3. Results

#### 6.3.1. Statistical Results

Table 10 summarizes the statistical results of 30 independent runs for EMPSO, BPSO, and BBA on the IEEE 30-bus, 39-bus, 57-bus, and 118-bus systems. Table 11 presents the optimal PMU locations identified by EMPSO on different IEEE test systems.

EMPSO consistently outperforms both BPSO and BBA across all test systems in terms of optimal solution quality. For the IEEE 30-bus system, EMPSO matches BPSO’s best result (10 PMUs) and significantly outperforms BBA (11 PMUs). While BPSO shows a marginally better average (10.23 vs. 10.50) and standard deviation on this smaller system, EMPSO maintains consistent worst-case performance.

As system complexity increases, EMPSO’s advantages become more pronounced. For the IEEE 39-bus system, EMPSO achieves a superior best-case solution (13 PMUs) compared to both BPSO (15 PMUs) and BBA (17 PMUs). This performance advantage extends to the larger systems, where EMPSO demonstrates remarkable scalability. Particularly notable is its performance on the IEEE 118-bus system, where EMPSO achieves a best-case solution of 44 PMUs—significantly better than BPSO’s 51 PMUs and vastly superior to BBA’s 61 PMUs.

The statistical results clearly demonstrate EMPSO’s robustness in maintaining solution quality across multiple runs. EMPSO strikes an optimal balance between exploration and exploitation, enabling it to escape local optima and find better solutions while maintaining reasonable consistency, especially given the complexity of the search space in larger systems.

#### 6.3.2. Convergence Analysis

**Figure 6:**
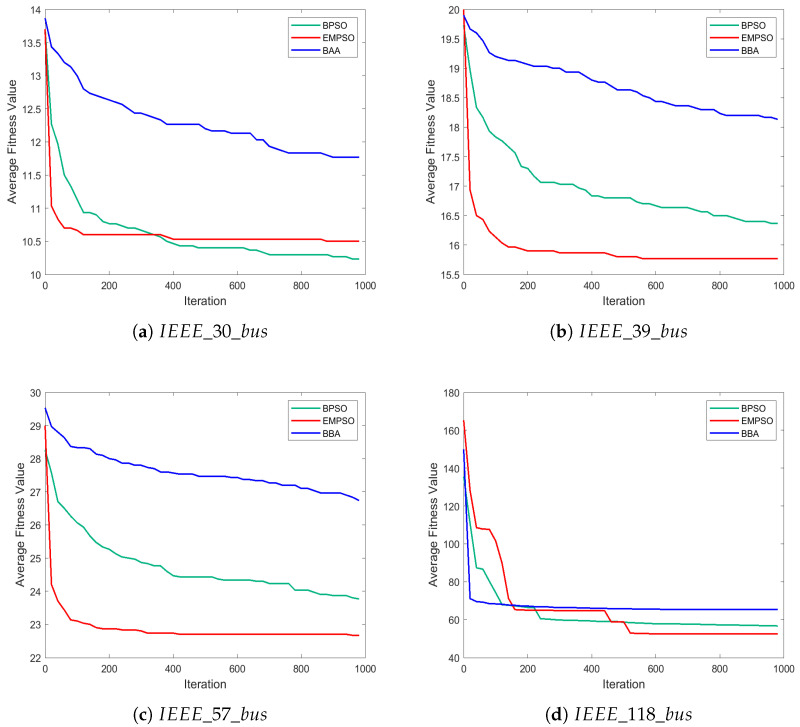
Convergence curves of EMPSO, BPSO, and BBA on the OPP problem.

Figure 6 illustrates the convergence behavior of EMPSO, BPSO, and BBA across the IEEE 30-, 39-, 57-, and 118-bus systems, revealing distinct performance differences. Overall, EMPSO achieves both faster convergence and higher solution quality than the other algorithms.

In the early iterations, EMPSO rapidly decreases the objective value, quickly approaching high-quality regions. BPSO also converges fast initially but tends to stagnate later due to premature convergence. In contrast, BBA exhibits the slowest convergence, with limited improvement over time, reflecting weak global exploration capability.

As the system size increases, EMPSO’s advantage becomes more evident. In the 118-bus system, EMPSO continues improving and reaches the best final solution, whereas BPSO and BBA stop making significant progress early. This demonstrates EMPSO’s scalability and robustness for large-scale, complex optimization problems, attributed to its enhanced exploration and exemplar-guided learning mechanisms that help avoid local entrapment.

These convergence trends are consistent with the statistical results, confirming EMPSO’s superior performance in the optimal PMU placement task. In particular, the algorithm effectively maintains population diversity and achieves a stable balance between exploration and exploitation throughout the optimization process.

### 6.4. Summary

In summary, the experimental study on the OPP problem demonstrates the superior performance, robustness, and scalability of the proposed EMPSO algorithm across multiple IEEE benchmark systems. EMPSO consistently requires fewer PMUs than both BPSO and BBA while maintaining a lower standard deviation, indicating its stability across independent runs. The integration of exemplar learning, memory retrieval, and adaptive position updating effectively enhances search efficiency and solution quality under discrete encoding.

Convergence analyses further reveal that EMPSO not only achieves faster descent in early iterations but also sustains improvement in the later stages, thereby avoiding the premature stagnation typically observed in competing algorithms. This advantage becomes increasingly pronounced as the network scale grows from 30 to 118 buses, validating EMPSO’s capability to handle large and complex search spaces efficiently.

Overall, EMPSO maintains a stable trade-off between exploration and exploitation, preserves population diversity, and exhibits strong generalization to binary optimization tasks. These findings substantiate EMPSO as a promising and extensible framework for large-scale engineering optimization problems such as PMU placement in power systems.

## 7. Conclusions and Future Work

This paper introduced EMPSO, a biologically inspired PSO variant that integrates EEL, SMR, and APU) mechanisms to overcome the limitations of premature convergence and insufficient adaptability in conventional PSO. By combining exemplar-driven learning, memory-based knowledge reuse, and fitness-dependent behavioral differentiation, EMPSO establishes a unified framework that enhances swarm intelligence through self-adaptive knowledge evolution.

Extensive experiments on the CEC2017 and CEC2022 benchmark suites confirm that EMPSO achieves superior convergence accuracy and stability across diverse problem landscapes. Its advantage becomes particularly pronounced in large-scale and multimodal scenarios, demonstrating that hierarchical exemplar learning and recency-weighted memory retrieval effectively sustain diversity and prevent stagnation. Applications to engineering design and PMU placement further validate EMPSO’s practicality, achieving consistent improvements in solution quality, robustness, and computational efficiency.

For future research, several directions are worth pursuing. First, the current memory retrieval process relies on a fixed recency decay; designing an adaptive forgetting mechanism or integrating reinforcement learning could yield more responsive memory management. Second, the exemplar aggregation in EEL may be extended through data-driven weighting, where the influence of elites is adaptively estimated using landscape metrics or clustering information. Third, probabilistic priors and Bayesian processing can provide more robust uncertainty quantification and may complement EMPSO. Therefore, combining probabilistic priors or surrogate-based uncertainty modeling with EMPSO could be a promising direction for future research. Finally, future studies could extend EMPSO to dynamic, multiobjective, and high-dimensional optimization, or embed it in hybrid systems such as deep learning training, energy management, or industrial scheduling to explore its scalability and domain adaptability.

Overall, this study contributes a novel perspective on strengthening swarm intelligence via exemplar-driven knowledge reuse and adaptive evolution, and we expect it to inspire the development of more resilient and knowledge-intensive swarm optimizers.

## Figures and Tables

**Table 1:** Parameter settings of EMPSO and the peer algorithms.

Algorithm	Other Parameters
EMPSO	ω=0.9→0.4, $c_{1}=c_{2}=1.5$, λ=0.2
KLDE [53]	LR=0.2,EP=10,F=0.5,CR=0.9
MPSO [54]	ω=0.9→0.4, $c_{1}=c_{2}=2$
AWPSO [29]	ω=0.9→0.4, $c_{1}=c_{2}=2,a\;=0.000035m,b=0.5,c=0,d=1.5$
PECSO [17]	η=0.5,α=1
WOA [6]	*a* decreases linearly: a∈ [0,2], b=1, $l=(a_{2}-1)\cdot \mathrm{rand}+1$
PSO [4]	ω=0.9→0.4, $c_{1}=c_{2}=1.5$

**Table 2:** Experimental results on the CEC2017 benchmark functions.

Funtion		EMPSO	KLDE	MPSO	AWPSO	PECSO	WOA	PSO
F1	Mean	6.87 $\times \;10^{2}$	4.74 $\times \;10^{3}$	3.66 $\times \;10^{8}$	4.50 $\times \;10^{6}$	3.60 $\times \;10^{3}$	3.00 $\times \;10^{8}$	7.69 $\times \;10^{9}$
	Std	5.36 $\times \;10^{2}$	4.72 $\times \;10^{3}$	3.25 $\times \;10^{8}$	4.94 $\times \;10^{6}$	1.37 $\times \;10^{3}$	7.15 $\times \;10^{7}$	2.96 $\times \;10^{9}$
	Rank	1	3	6	4	2	5	7
F3	Mean	1.04 $\times \;10^{4}$	8.77 $\times \;10^{4}$	6.76 $\times \;10^{4}$	4.99 $\times \;10^{3}$	5.17 $\times \;10^{3}$	2.09 $\times \;10^{5}$	4.53 $\times \;10^{4}$
	Std	2.89 $\times \;10^{3}$	1.95 $\times \;10^{4}$	1.78 $\times \;10^{4}$	2.52 $\times \;10^{3}$	1.28 $\times \;10^{3}$	3.52 $\times \;10^{4}$	1.53 $\times \;10^{4}$
	Rank	3	6	5	1	2	7	4
F4	Mean	4.87 $\times \;10^{2}$	4.86 $\times \;10^{2}$	5.54 $\times \;10^{2}$	6.25 $\times \;10^{2}$	4.92 $\times \;10^{2}$	6.42 $\times \;10^{2}$	1.29 $\times \;10^{3}$
	Std	1.48	3.03	8.55 $\times \;10^{1}$	5.64 $\times \;10^{1}$	1.13 $\times \;10^{1}$	4.63 $\times \;10^{1}$	4.42 $\times \;10^{2}$
	Rank	2	1	4	5	3	6	7
F5	Mean	5.12 $\times \;10^{2}$	6.35 $\times \;10^{2}$	6.46 $\times \;10^{2}$	6.20 $\times \;10^{2}$	6.28 $\times \;10^{2}$	7.75 $\times \;10^{2}$	6.33 $\times \;10^{2}$
	Std	2.77	4.46 $\times \;10^{1}$	5.19 $\times \;10^{1}$	3.60 $\times \;10^{1}$	3.91	4.30 $\times \;10^{1}$	2.21 $\times \;10^{1}$
	Rank	1	5	6	2	3	7	4
F6	Mean	6.00 $\times \;10^{2}$	6.00 $\times \;10^{2}$	6.06 $\times \;10^{2}$	6.18 $\times \;10^{2}$	6.11 $\times \;10^{2}$	6.69 $\times \;10^{2}$	6.17 $\times \;10^{2}$
	Std	1.20 $\times \;10^{-2}$	1.08 $\times \;10^{-4}$	1.85	6.11	5.57 $\times \;10^{-1}$	5.83	5.35
	Rank	2	1	3	6	4	7	5
F7	Mean	7.45 $\times \;10^{2}$	8.91 $\times \;10^{2}$	9.11 $\times \;10^{2}$	9.12 $\times \;10^{2}$	9.28 $\times \;10^{2}$	1.21 $\times \;10^{3}$	9.21 $\times \;10^{2}$
	Std	2.86	1.29 $\times \;10^{1}$	3.66 $\times \;10^{1}$	5.66 $\times \;10^{1}$	6.07	8.11 $\times \;10^{1}$	5.48 $\times \;10^{1}$
	Rank	1	2	3	4	6	7	5
F8	Mean	8.12 $\times \;10^{2}$	9.29 $\times \;10^{2}$	9.41 $\times \;10^{2}$	9.21 $\times \;10^{2}$	9.02 $\times \;10^{2}$	9.87 $\times \;10^{2}$	9.24 $\times \;10^{2}$
	Std	1.85	4.25 $\times \;10^{1}$	4.52 $\times \;10^{1}$	3.14 $\times \;10^{1}$	4.00	2.85 $\times \;10^{1}$	2.68 $\times \;10^{1}$
	Rank	1	5	6	3	2	7	4
F9	Mean	9.01 $\times \;10^{2}$	9.00 $\times \;10^{2}$	1.04 $\times \;10^{3}$	2.11 $\times \;10^{3}$	2.82 $\times \;10^{3}$	7.63 $\times \;10^{3}$	3.66 $\times \;10^{3}$
	Std	3.97 $\times \;10^{-1}$	8.29 $\times \;10^{-2}$	1.41 $\times \;10^{2}$	8.22 $\times \;10^{2}$	1.22 $\times \;10^{1}$	1.76 $\times \;10^{3}$	1.13 $\times \;10^{3}$
	Rank	2	1	3	4	5	7	6
F10	Mean	3.52 $\times \;10^{3}$	7.31 $\times \;10^{3}$	7.61 $\times \;10^{3}$	4.94 $\times \;10^{3}$	4.39 $\times \;10^{3}$	6.35 $\times \;10^{3}$	4.72 $\times \;10^{3}$
	Std	4.53 $\times \;10^{2}$	8.62 $\times \;10^{2}$	8.15 $\times \;10^{2}$	7.49 $\times \;10^{2}$	2.04 $\times \;10^{3}$	5.54 $\times \;10^{2}$	3.77 $\times \;10^{2}$
	Rank	1	6	7	4	2	5	3
F11	Mean	1.14 $\times \;10^{3}$	1.18 $\times \;10^{3}$	1.34 $\times \;10^{3}$	1.37 $\times \;10^{3}$	1.23 $\times \;10^{3}$	3.09 $\times \;10^{3}$	1.44 $\times \;10^{3}$
	Std	1.83 $\times \;10^{1}$	3.17 $\times \;10^{1}$	2.30 $\times \;10^{2}$	8.24 $\times \;10^{1}$	5.75 $\times \;10^{1}$	8.02 $\times \;10^{2}$	7.67 $\times \;10^{1}$
	Rank	1	2	4	5	3	7	6
F12	Mean	3.16 $\times \;10^{4}$	5.26 $\times \;10^{4}$	5.23 $\times \;10^{6}$	2.58 $\times \;10^{7}$	2.12 $\times \;10^{6}$	9.13 $\times \;10^{7}$	2.36 $\times \;10^{8}$
	Std	1.21 $\times \;10^{4}$	2.15 $\times \;10^{4}$	5.70 $\times \;10^{6}$	3.61 $\times \;10^{7}$	2.50 $\times \;10^{4}$	4.94 $\times \;10^{7}$	2.15 $\times \;10^{8}$
	Rank	1	2	4	5	3	6	7
F13	Mean	8.06 $\times \;10^{3}$	1.44 $\times \;10^{3}$	3.26 $\times \;10^{4}$	3.62 $\times \;10^{4}$	1.27 $\times \;10^{4}$	3.65 $\times \;10^{5}$	1.81 $\times \;10^{6}$
	Std	5.92 $\times \;10^{3}$	1.85 $\times \;10^{1}$	6.69 $\times \;10^{4}$	3.61 $\times \;10^{4}$	1.20 $\times \;10^{4}$	1.42 $\times \;10^{5}$	2.14 $\times \;10^{6}$
	Rank	2	1	4	5	3	6	7
F14	Mean	6.00 $\times \;10^{3}$	1.48 $\times \;10^{3}$	3.59 $\times \;10^{5}$	4.37 $\times \;10^{3}$	3.42 $\times \;10^{4}$	6.36 $\times \;10^{5}$	3.35 $\times \;10^{4}$
	Std	3.40 $\times \;10^{3}$	6.47	3.79 $\times \;10^{5}$	7.27 $\times \;10^{3}$	2.58 $\times \;10^{3}$	4.56 $\times \;10^{5}$	2.63 $\times \;10^{4}$
	Rank	3	1	6	2	5	7	4
F15	Mean	1.97 $\times \;10^{3}$	1.56 $\times \;10^{3}$	3.27 $\times \;10^{3}$	1.28 $\times \;10^{4}$	5.52 $\times \;10^{3}$	1.34 $\times \;10^{5}$	3.57 $\times \;10^{4}$
	Std	3.42 $\times \;10^{2}$	6.64	1.85 $\times \;10^{3}$	1.37 $\times \;10^{4}$	1.25 $\times \;10^{3}$	4.36 $\times \;10^{4}$	1.81 $\times \;10^{4}$
	Rank	2	1	3	5	4	6	7
F16	Mean	1.95 $\times \;10^{3}$	2.16 $\times \;10^{3}$	2.82 $\times \;10^{3}$	2.52 $\times \;10^{3}$	2.66 $\times \;10^{3}$	3.72 $\times \;10^{3}$	2.74 $\times \;10^{3}$
	Std	1.81 $\times \;10^{2}$	3.04 $\times \;10^{2}$	3.85 $\times \;10^{2}$	2.71 $\times \;10^{2}$	2.45 $\times \;10^{2}$	2.42 $\times \;10^{2}$	2.34 $\times \;10^{2}$
	Rank	1	2	6	3	4	7	5
F17	Mean	1.83 $\times \;10^{3}$	1.84 $\times \;10^{3}$	2.21 $\times \;10^{3}$	2.19 $\times \;10^{3}$	2.25 $\times \;10^{3}$	2.49 $\times \;10^{3}$	2.31 $\times \;10^{3}$
	Std	6.35 $\times \;10^{1}$	1.15 $\times \;10^{2}$	2.35 $\times \;10^{2}$	2.05 $\times \;10^{2}$	5.12 $\times \;10^{1}$	1.61 $\times \;10^{2}$	1.52 $\times \;10^{2}$
	Rank	1	2	4	3	5	7	6
F18	Mean	7.12 $\times \;10^{4}$	1.88 $\times \;10^{3}$	8.99 $\times \;10^{5}$	1.88 $\times \;10^{5}$	5.19 $\times \;10^{5}$	3.06 $\times \;10^{6}$	2.88 $\times \;10^{5}$
	Std	2.46 $\times \;10^{4}$	5.52	9.12 $\times \;10^{5}$	3.05 $\times \;10^{5}$	4.02 $\times \;10^{4}$	2.22 $\times \;10^{6}$	1.56 $\times \;10^{5}$
	Rank	2	1	6	3	5	7	4
F19	Mean	3.88 $\times \;10^{3}$	1.94 $\times \;10^{3}$	6.14 $\times \;10^{3}$	1.05 $\times \;10^{4}$	7.62 $\times \;10^{3}$	3.99 $\times \;10^{6}$	5.63 $\times \;10^{5}$
	Std	1.44 $\times \;10^{3}$	4.45	4.03 $\times \;10^{3}$	1.27 $\times \;10^{4}$	1.46 $\times \;10^{3}$	1.79 $\times \;10^{6}$	7.35 $\times \;10^{5}$
	Rank	2	1	3	5	4	7	6
F20	Mean	2.13 $\times \;10^{3}$	2.22 $\times \;10^{3}$	2.42 $\times \;10^{3}$	2.41 $\times \;10^{3}$	2.53 $\times \;10^{3}$	2.68 $\times \;10^{3}$	2.38 $\times \;10^{3}$
	Std	6.34 $\times \;10^{1}$	1.80 $\times \;10^{2}$	1.92 $\times \;10^{2}$	1.62 $\times \;10^{2}$	4.70 $\times \;10^{1}$	1.21 $\times \;10^{2}$	1.42 $\times \;10^{2}$
	Rank	1	2	5	4	6	7	3
F21	Mean	2.32 $\times \;10^{3}$	2.44 $\times \;10^{3}$	2.44 $\times \;10^{3}$	2.40 $\times \;10^{3}$	2.41 $\times \;10^{3}$	2.56 $\times \;10^{3}$	2.44 $\times \;10^{3}$
	Std	2.93	3.88 $\times \;10^{1}$	5.48 $\times \;10^{1}$	2.96 $\times \;10^{1}$	4.86	3.33 $\times \;10^{1}$	2.41 $\times \;10^{1}$
	Rank	1	5	6	2	3	7	4
F22	Mean	2.30 $\times \;10^{3}$	6.30 $\times \;10^{3}$	2.76 $\times \;10^{3}$	2.48 $\times \;10^{3}$	4.32 $\times \;10^{3}$	7.13 $\times \;10^{3}$	5.10 $\times \;10^{3}$
	Std	4.48 $\times \;10^{-1}$	3.35 $\times \;10^{3}$	1.55 $\times \;10^{3}$	7.78 $\times \;10^{2}$	1.68 $\times \;10^{3}$	1.22 $\times \;10^{3}$	1.24 $\times \;10^{3}$
	Rank	1	6	3	2	4	7	5
F23	Mean	2.75 $\times \;10^{3}$	2.75 $\times \;10^{3}$	2.75 $\times \;10^{3}$	2.76 $\times \;10^{3}$	2.83 $\times \;10^{3}$	3.03 $\times \;10^{3}$	2.93 $\times \;10^{3}$
	Std	2.16 $\times \;10^{1}$	5.58 $\times \;10^{1}$	4.94 $\times \;10^{1}$	2.91 $\times \;10^{1}$	1.88 $\times \;10^{1}$	5.74 $\times \;10^{1}$	5.39 $\times \;10^{1}$
	Rank	1	3	2	4	5	7	6
F24	Mean	2.90 $\times \;10^{3}$	2.96 $\times \;10^{3}$	2.95 $\times \;10^{3}$	2.91 $\times \;10^{3}$	3.01 $\times \;10^{3}$	3.14 $\times \;10^{3}$	3.12 $\times \;10^{3}$
	Std	1.46 $\times \;10^{1}$	4.98 $\times \;10^{1}$	6.20 $\times \;10^{1}$	3.03 $\times \;10^{1}$	1.33 $\times \;10^{1}$	6.90 $\times \;10^{1}$	4.81 $\times \;10^{1}$
	Rank	1	4	3	2	5	7	6
F25	Mean	2.89 $\times \;10^{3}$	2.89 $\times \;10^{3}$	3.01 $\times \;10^{3}$	2.95 $\times \;10^{3}$	2.90 $\times \;10^{3}$	3.03 $\times \;10^{3}$	3.05 $\times \;10^{3}$
	Std	1.58 $\times \;10^{-1}$	9.78 $\times \;10^{-2}$	3.69 $\times \;10^{1}$	2.88 $\times \;10^{1}$	1.63	2.03 $\times \;10^{1}$	9.33 $\times \;10^{1}$
	Rank	2	1	5	4	3	6	7
F26	Mean	4.33 $\times \;10^{3}$	4.37 $\times \;10^{3}$	4.51 $\times \;10^{3}$	4.37 $\times \;10^{3}$	5.64 $\times \;10^{3}$	7.23 $\times \;10^{3}$	6.19 $\times \;10^{3}$
	Std	1.57 $\times \;10^{2}$	5.58 $\times \;10^{2}$	1.11 $\times \;10^{3}$	1.09 $\times \;10^{3}$	4.07 $\times \;10^{2}$	1.06 $\times \;10^{3}$	6.54 $\times \;10^{2}$
	Rank	1	2	4	3	5	7	6
F27	Mean	3.25 $\times \;10^{3}$	3.20 $\times \;10^{3}$	3.26 $\times \;10^{3}$	3.28 $\times \;10^{3}$	3.20 $\times \;10^{3}$	3.35 $\times \;10^{3}$	3.31 $\times \;10^{3}$
	Std	1.60 $\times \;10^{1}$	7.64	2.53 $\times \;10^{1}$	2.39 $\times \;10^{1}$	1.47 $\times \;10^{1}$	4.56 $\times \;10^{1}$	3.13 $\times \;10^{1}$
	Rank	3	1	4	5	2	7	6
F28	Mean	3.20 $\times \;10^{3}$	3.20 $\times \;10^{3}$	3.42 $\times \;10^{3}$	3.34 $\times \;10^{3}$	3.25 $\times \;10^{3}$	3.41 $\times \;10^{3}$	4.03 $\times \;10^{3}$
	Std	3.50 $\times \;10^{1}$	4.02 $\times \;10^{1}$	7.14 $\times \;10^{1}$	5.92 $\times \;10^{1}$	3.99 $\times \;10^{1}$	2.45 $\times \;10^{1}$	4.22 $\times \;10^{2}$
	Rank	1	2	6	4	3	5	7
F29	Mean	3.48 $\times \;10^{3}$	3.51 $\times \;10^{3}$	3.76 $\times \;10^{3}$	3.99 $\times \;10^{3}$	3.72 $\times \;10^{3}$	4.77 $\times \;10^{3}$	3.87 $\times \;10^{3}$
	Std	8.93 $\times \;10^{1}$	1.02 $\times \;10^{2}$	2.12 $\times \;10^{2}$	2.01 $\times \;10^{2}$	1.31 $\times \;10^{2}$	2.40 $\times \;10^{2}$	1.82 $\times \;10^{2}$
	Rank	1	2	4	6	3	7	5
F30	Mean	6.86 $\times \;10^{3}$	6.36 $\times \;10^{3}$	9.88 $\times \;10^{4}$	9.89 $\times \;10^{5}$	7.10 $\times \;10^{3}$	1.13 $\times \;10^{7}$	1.57 $\times \;10^{6}$
	Std	8.74 $\times \;10^{2}$	5.86 $\times \;10^{2}$	1.91 $\times \;10^{5}$	1.15 $\times \;10^{6}$	1.83 $\times \;10^{3}$	6.26 $\times \;10^{6}$	1.08 $\times \;10^{6}$
	Rank	2	1	4	5	3	7	6
	Mean Rank	1.52	2.48	4.45	3.79	3.69	6.62	5.45
	Final Rank	1	2	5	4	3	7	6
	Time Taken	2231.80	48859.42	1935.85	1789.36	1801.12	1207.04	1315.51

**Table 3:** Wilcoxon rank-sum test results of EMPSO against 6 representative algorithms on the CEC2017 benchmark functions (significance level α=0.05).

Function	KLDE	MPSO	AWPSO	PECSO	WOA	PSO
F1	+	+	+	+	+	+
F3	+	+	−	−	+	+
F4	≈	+	+	+	+	+
F5	+	+	+	+	+	+
F6	−	+	+	+	+	+
F7	+	+	+	+	+	+
F8	+	+	+	+	+	+
F9	−	+	+	+	+	+
F10	+	+	+	+	+	+
F11	+	+	+	+	+	+
F12	+	+	+	+	+	+
F13	−	+	+	+	+	+
F14	−	+	−	+	+	+
F15	−	+	+	+	+	+
F16	+	+	+	+	+	+
F17	+	+	+	+	+	+
F18	−	+	+	+	+	+
F19	−	+	+	+	+	+
F20	+	+	+	+	+	+
F21	+	+	+	+	+	+
F22	+	+	+	+	+	+
F23	+	+	+	+	+	+
F24	+	+	≈	+	+	+
F25	-	+	+	+	+	+
F26	+	+	+	+	+	+
F27	−	+	+	−	+	+
F28	≈	+	+	+	+	+
F29	+	+	+	+	+	+
F30	−	+	+	+	+	+
Better	17	29	26	27	29	29
Similar	2	0	1	0	0	0
Worse	10	0	2	2	0	0

**Table 4:** Experimental results on the CEC2022 benchmark functions.

Funtion		EMPSO	KLDE	MPSO	AWPSO	PECSO	WOA	PSO
F1	Mean	3.00 $\times \;10^{2}$	3.64 $\times \;10^{2}$	4.59 $\times \;10^{2}$	3.20 $\times \;10^{2}$	3.02 $\times \;10^{2}$	4.96 $\times \;10^{3}$	3.01 $\times \;10^{2}$
	Std	5.71 $\times \;10^{-3}$	9.95 $\times \;10^{1}$	6.54 $\times \;10^{1}$	1.21 $\times \;10^{1}$	1.26	1.80 $\times \;10^{3}$	1.22
	Rank	1	5	6	4	3	7	2
F2	Mean	4.49 $\times \;10^{2}$	4.49 $\times \;10^{2}$	4.45 $\times \;10^{2}$	4.57 $\times \;10^{2}$	4.29 $\times \;10^{2}$	4.76 $\times \;10^{2}$	4.90 $\times \;10^{2}$
	Std	7.65 $\times \;10^{-1}$	1.32	1.15 $\times \;10^{1}$	4.95	1.96 $\times \;10^{1}$	1.42 $\times \;10^{1}$	3.41 $\times \;10^{1}$
	Rank	3	4	2	5	1	6	7
F3	Mean	6.00 $\times \;10^{2}$	6.00 $\times \;10^{2}$	6.00 $\times \;10^{2}$	6.00 $\times \;10^{2}$	6.01 $\times \;10^{2}$	6.53 $\times \;10^{2}$	6.07 $\times \;10^{2}$
	Std	9.57 $\times \;10^{-2}$	9.25 $\times \;10^{-6}$	1.51 $\times \;10^{-1}$	8.14 $\times \;10^{-4}$	1.09	7.42	3.76
	Rank	3	1	4	2	5	7	6
F4	Mean	8.06 $\times \;10^{2}$	8.52 $\times \;10^{2}$	8.26 $\times \;10^{2}$	8.33 $\times \;10^{2}$	8.47 $\times \;10^{2}$	8.86 $\times \;10^{2}$	8.51 $\times \;10^{2}$
	Std	1.58	2.85 $\times \;10^{1}$	6.37	5.59	9.95	1.64 $\times \;10^{1}$	8.63
	Rank	1	6	2	3	4	7	5
F5	Mean	9.00 $\times \;10^{2}$	9.00 $\times \;10^{2}$	9.01 $\times \;10^{2}$	9.12 $\times \;10^{2}$	1.27 $\times \;10^{3}$	2.72 $\times \;10^{3}$	1.06 $\times \;10^{3}$
	Std	1.01 $\times \;10^{-13}$	2.83 $\times \;10^{-2}$	8.93 $\times \;10^{-1}$	8.39	1.79 $\times \;10^{2}$	4.98 $\times \;10^{2}$	1.39 $\times \;10^{2}$
	Rank	1	2	3	4	6	7	5
F6	Mean	2.40 $\times \;10^{3}$	1.83 $\times \;10^{3}$	2.81 $\times \;10^{3}$	4.24 $\times \;10^{3}$	2.87 $\times \;10^{3}$	7.67 $\times \;10^{3}$	8.85 $\times \;10^{3}$
	Std	4.14 $\times \;10^{2}$	1.23 $\times \;10^{1}$	7.32 $\times \;10^{2}$	2.23 $\times \;10^{3}$	8.52 $\times \;10^{2}$	3.51 $\times \;10^{3}$	7.24 $\times \;10^{3}$
	Rank	2	1	3	5	4	6	7
F7	Mean	2.02 $\times \;10^{3}$	2.04 $\times \;10^{3}$	2.04 $\times \;10^{3}$	2.02 $\times \;10^{3}$	2.05 $\times \;10^{3}$	2.15 $\times \;10^{3}$	2.04 $\times \;10^{3}$
	Std	1.86	5.46	6.25	1.94	1.32 $\times \;10^{1}$	2.86 $\times \;10^{1}$	9.97
	Rank	1	3	4	2	6	7	5
F8	Mean	2.22 $\times \;10^{3}$	2.23 $\times \;10^{3}$	2.23 $\times \;10^{3}$	2.22 $\times \;10^{3}$	2.23 $\times \;10^{3}$	2.25 $\times \;10^{3}$	2.23 $\times \;10^{3}$
	Std	7.30 $\times \;10^{-1}$	1.75	1.91	4.73 $\times \;10^{-1}$	7.61	9.23	4.50
	Rank	2	3	4	1	6	7	5
F9	Mean	2.48 $\times \;10^{3}$	2.48 $\times \;10^{3}$	2.48 $\times \;10^{3}$	2.48 $\times \;10^{3}$	2.47 $\times \;10^{3}$	2.49 $\times \;10^{3}$	2.52 $\times \;10^{3}$
	Std	3.27 $\times \;10^{-13}$	9.27 $\times \;10^{-12}$	5.54 $\times \;10^{-1}$	4.79 $\times \;10^{-1}$	3.09	8.02	2.44 $\times \;10^{1}$
	Rank	1	2	4	3	5	6	7
F10	Mean	2.53 $\times \;10^{3}$	2.82 $\times \;10^{3}$	2.52 $\times \;10^{3}$	2.53 $\times \;10^{3}$	2.59 $\times \;10^{3}$	3.72 $\times \;10^{3}$	2.76 $\times \;10^{3}$
	Std	5.40 $\times \;10^{1}$	6.89 $\times \;10^{2}$	4.15 $\times \;10^{1}$	6.03 $\times \;10^{1}$	8.86 $\times \;10^{1}$	1.02 $\times \;10^{3}$	4.26 $\times \;10^{2}$
	Rank	2	6	1	3	4	7	5
F11	Mean	2.90 $\times \;10^{3}$	2.93 $\times \;10^{3}$	2.94 $\times \;10^{3}$	2.89 $\times \;10^{3}$	2.89 $\times \;10^{3}$	2.97 $\times \;10^{3}$	3.90 $\times \;10^{3}$
	Std	1.44 $\times \;10^{-12}$	4.83 $\times \;10^{1}$	7.60 $\times \;10^{1}$	5.48 $\times \;10^{1}$	5.48 $\times \;10^{1}$	1.26 $\times \;10^{1}$	4.77 $\times \;10^{2}$
	Rank	3	4	5	1	2	6	7
F12	Mean	2.96 $\times \;10^{3}$	2.94 $\times \;10^{3}$	2.96 $\times \;10^{3}$	2.96 $\times \;10^{3}$	2.90 $\times \;10^{3}$	3.00 $\times \;10^{3}$	2.97 $\times \;10^{3}$
	Std	1.22 $\times \;10^{1}$	7.77	7.27	6.71	1.50 $\times \;10^{-4}$	2.26 $\times \;10^{1}$	1.65 $\times \;10^{1}$
	Rank	5	2	3	4	1	7	6
	Mean Rank	2.08	3.25	3.42	3.08	3.92	6.67	5.58
	Final Rank	1	3	4	2	5	7	6
	Time Taken	564.85	12464.14	488.85	448.48	461.57	305.58	328.66

**Table 5:** Sensitivity analysis of parameter λ on the CEC2017 benchmark functions.

Function	λ=0	λ=0.1	λ=0.2	λ=0.3	λ=0.5
F1	2.15 $\times \;10^{3}$	2.58 $\times \;10^{3}$	1.61 $\times \;10^{3}$	1.90 $\times \;10^{3}$	2.28 $\times \;10^{3}$
F3	2.18 $\times \;10^{3}$	5.22 $\times \;10^{2}$	4.52 $\times \;10^{2}$	4.88 $\times \;10^{2}$	5.13 $\times \;10^{2}$
F4	4.88 $\times \;10^{2}$	4.90 $\times \;10^{2}$	4.88 $\times \;10^{2}$	4.93 $\times \;10^{2}$	4.93 $\times \;10^{2}$
F5	5.27 $\times \;10^{2}$	5.32 $\times \;10^{2}$	5.32 $\times \;10^{2}$	5.33 $\times \;10^{2}$	5.33 $\times \;10^{2}$
F6	6.01 $\times \;10^{2}$	6.01 $\times \;10^{2}$	6.01 $\times \;10^{2}$	6.01 $\times \;10^{2}$	6.01 $\times \;10^{2}$
F7	7.67 $\times \;10^{2}$	7.61 $\times \;10^{2}$	7.63 $\times \;10^{2}$	7.62 $\times \;10^{2}$	7.64 $\times \;10^{2}$
F8	8.24 $\times \;10^{2}$	8.30 $\times \;10^{2}$	8.32 $\times \;10^{2}$	8.31 $\times \;10^{2}$	8.31 $\times \;10^{2}$
F9	9.38 $\times \;10^{2}$	9.09 $\times \;10^{2}$	9.08 $\times \;10^{2}$	9.09 $\times \;10^{2}$	9.08 $\times \;10^{2}$
F10	5.95 $\times \;10^{3}$	3.53 $\times \;10^{3}$	3.38 $\times \;10^{3}$	3.40 $\times \;10^{3}$	3.54 $\times \;10^{3}$
F11	1.26 $\times \;10^{3}$	1.24 $\times \;10^{3}$	1.23 $\times \;10^{3}$	1.23 $\times \;10^{3}$	1.24 $\times \;10^{3}$
F12	7.57 $\times \;10^{4}$	6.92 $\times \;10^{4}$	4.89 $\times \;10^{4}$	7.50 $\times \;10^{4}$	7.24 $\times \;10^{4}$
F13	1.45 $\times \;10^{4}$	1.39 $\times \;10^{4}$	1.11 $\times \;10^{4}$	1.68 $\times \;10^{4}$	2.09 $\times \;10^{4}$
F14	4.66 $\times \;10^{3}$	3.65 $\times \;10^{3}$	3.57 $\times \;10^{3}$	3.79 $\times \;10^{3}$	3.71 $\times \;10^{3}$
F15	3.19 $\times \;10^{3}$	3.09 $\times \;10^{3}$	2.59 $\times \;10^{3}$	3.07 $\times \;10^{3}$	3.16 $\times \;10^{3}$
F16	2.09 $\times \;10^{3}$	2.06 $\times \;10^{3}$	2.03 $\times \;10^{3}$	2.09 $\times \;10^{3}$	2.11 $\times \;10^{3}$
F17	1.86 $\times \;10^{3}$	1.88 $\times \;10^{3}$	1.86 $\times \;10^{3}$	1.89 $\times \;10^{3}$	1.89 $\times \;10^{3}$
F18	8.31 $\times \;10^{4}$	7.47 $\times \;10^{4}$	5.67 $\times \;10^{4}$	7.09 $\times \;10^{4}$	7.32 $\times \;10^{4}$
F19	4.21 $\times \;10^{3}$	3.57 $\times \;10^{3}$	3.90 $\times \;10^{3}$	3.98 $\times \;10^{3}$	4.14 $\times \;10^{3}$
F20	2.18 $\times \;10^{3}$	2.15 $\times \;10^{3}$	2.16 $\times \;10^{3}$	2.17 $\times \;10^{3}$	2.18 $\times \;10^{3}$
F21	2.33 $\times \;10^{3}$	2.34 $\times \;10^{3}$	2.34 $\times \;10^{3}$	2.34 $\times \;10^{3}$	2.34 $\times \;10^{3}$
F22	3.18 $\times \;10^{3}$	3.24 $\times \;10^{3}$	3.15 $\times \;10^{3}$	3.09 $\times \;10^{3}$	3.11 $\times \;10^{3}$
F23	2.74 $\times \;10^{3}$	2.76 $\times \;10^{3}$	2.74 $\times \;10^{3}$	2.79 $\times \;10^{3}$	2.80 $\times \;10^{3}$
F24	2.90 $\times \;10^{3}$	2.92 $\times \;10^{3}$	2.93 $\times \;10^{3}$	2.95 $\times \;10^{3}$	2.97 $\times \;10^{3}$
F25	2.89 $\times \;10^{3}$	2.89 $\times \;10^{3}$	2.89 $\times \;10^{3}$	2.89 $\times \;10^{3}$	2.89 $\times \;10^{3}$
F26	4.54 $\times \;10^{3}$	4.65 $\times \;10^{3}$	4.65 $\times \;10^{3}$	4.75 $\times \;10^{3}$	4.70 $\times \;10^{3}$
F27	3.25 $\times \;10^{3}$	3.27 $\times \;10^{3}$	3.25 $\times \;10^{3}$	3.28 $\times \;10^{3}$	3.27 $\times \;10^{3}$
F28	3.24 $\times \;10^{3}$	3.23 $\times \;10^{3}$	3.23 $\times \;10^{3}$	3.24 $\times \;10^{3}$	3.23 $\times \;10^{3}$
F29	3.64 $\times \;10^{3}$	3.59 $\times \;10^{3}$	3.55 $\times \;10^{3}$	3.60 $\times \;10^{3}$	3.61 $\times \;10^{3}$
F30	7.45 $\times \;10^{3}$	7.75 $\times \;10^{3}$	7.74 $\times \;10^{3}$	7.32 $\times \;10^{3}$	7.12 $\times \;10^{3}$

**Table 6:** Ablation study results of EMPSO and its variants on the CEC2017 functions.

Funtion		EMPSO	w/o_High	w/o_Middle	w/o_Low	PSO
F1	Mean	1.61 $\times \;10^{3}$	1.52 $\times \;10^{9}$	3.85 $\times \;10^{3}$	2.12 $\times \;10^{3}$	7.69 $\times \;10^{9}$
	Std	1.56 $\times \;10^{3}$	1.29 $\times \;10^{9}$	3.66 $\times \;10^{3}$	1.85 $\times \;10^{3}$	2.96 $\times \;10^{9}$
	Rank	1	4	3	2	5
F3	Mean	4.52 $\times \;10^{2}$	1.87 $\times \;10^{3}$	3.31 $\times \;10^{3}$	2.38 $\times \;10^{3}$	4.53 $\times \;10^{4}$
	Std	2.03 $\times \;10^{2}$	2.02 $\times \;10^{3}$	1.59 $\times \;10^{3}$	1.55 $\times \;10^{3}$	1.53 $\times \;10^{4}$
	Rank	1	2	4	3	5
F5	Mean	5.32 $\times \;10^{2}$	5.68 $\times \;10^{2}$	5.33 $\times \;10^{2}$	5.25 $\times \;10^{2}$	6.33 $\times \;10^{2}$
	Std	5.12	1.57 $\times \;10^{1}$	6.21	4.95	2.21 $\times \;10^{1}$
	Rank	2	4	3	1	5
F7	Mean	7.63 $\times \;10^{2}$	8.02 $\times \;10^{2}$	7.65 $\times \;10^{2}$	7.68 $\times \;10^{2}$	9.21 $\times \;10^{2}$
	Std	4.52	2.03 $\times \;10^{1}$	5.22	7.02	5.48 $\times \;10^{1}$
	Rank	1	4	2	3	5
F9	Mean	9.08 $\times \;10^{2}$	1.52 $\times \;10^{3}$	9.15 $\times \;10^{2}$	9.34 $\times \;10^{2}$	3.66 $\times \;10^{3}$
	Std	5.01	3.14 $\times \;10^{2}$	8.33	2.09 $\times \;10^{1}$	1.13 $\times \;10^{3}$
	Rank	1	4	2	3	5
F11	Mean	1.23 $\times \;10^{3}$	1.41 $\times \;10^{3}$	1.25 $\times \;10^{3}$	1.26 $\times \;10^{3}$	1.44 $\times \;10^{3}$
	Std	5.27 $\times \;10^{1}$	7.62 $\times \;10^{1}$	3.46 $\times \;10^{1}$	4.22 $\times \;10^{1}$	7.67 $\times \;10^{1}$
	Rank	1	4	2	3	5
F13	Mean	1.11 $\times \;10^{4}$	1.77 $\times \;10^{6}$	1.77 $\times \;10^{4}$	1.68 $\times \;10^{4}$	1.81 $\times \;10^{6}$
	Std	6.59 $\times \;10^{3}$	2.32 $\times \;10^{6}$	1.25 $\times \;10^{4}$	1.21 $\times \;10^{4}$	2.14 $\times \;10^{6}$
	Rank	1	4	3	2	5
F15	Mean	2.59 $\times \;10^{3}$	3.31 $\times \;10^{4}$	3.05 $\times \;10^{3}$	3.60 $\times \;10^{3}$	3.57 $\times \;10^{4}$
	Std	5.48 $\times \;10^{2}$	2.10 $\times \;10^{4}$	1.08 $\times \;10^{3}$	1.91 $\times \;10^{3}$	1.81 $\times \;10^{4}$
	Rank	1	4	2	3	5
F17	Mean	1.86 $\times \;10^{3}$	2.14 $\times \;10^{3}$	1.89 $\times \;10^{3}$	1.89 $\times \;10^{3}$	2.31 $\times \;10^{3}$
	Std	5.94 $\times \;10^{1}$	1.54 $\times \;10^{2}$	6.42 $\times \;10^{1}$	6.77 $\times \;10^{1}$	1.52 $\times \;10^{2}$
	Rank	1	4	2	3	5
F19	Mean	3.90 $\times \;10^{3}$	2.04 $\times \;10^{5}$	4.43 $\times \;10^{3}$	4.46 $\times \;10^{3}$	5.63 $\times \;10^{5}$
	Std	1.48 $\times \;10^{3}$	2.09 $\times \;10^{5}$	1.91 $\times \;10^{3}$	1.92 $\times \;10^{3}$	7.35 $\times \;10^{5}$
	Rank	1	2	3	4	5
F21	Mean	2.34 $\times \;10^{3}$	2.37 $\times \;10^{3}$	2.34 $\times \;10^{3}$	2.34 $\times \;10^{3}$	2.44 $\times \;10^{3}$
	Std	7.16	1.10 $\times \;10^{1}$	7.72	7.31	2.41 $\times \;10^{1}$
	Rank	1	4	3	2	5
F23	Mean	2.74 $\times \;10^{3}$	2.82 $\times \;10^{3}$	2.77 $\times \;10^{3}$	2.74 $\times \;10^{3}$	2.93 $\times \;10^{3}$
	Std	2.03 $\times \;10^{1}$	3.10 $\times \;10^{1}$	2.59 $\times \;10^{1}$	2.23 $\times \;10^{1}$	5.39 $\times \;10^{1}$
	Rank	1	4	3	2	5
F25	Mean	2.89 $\times \;10^{3}$	2.95 $\times \;10^{3}$	2.89 $\times \;10^{3}$	2.89 $\times \;10^{3}$	3.05 $\times \;10^{3}$
	Std	1.07	4.45 $\times \;10^{1}$	1.30	1.79	9.33 $\times \;10^{1}$
	Rank	1	4	2	3	5
F27	Mean	3.25 $\times \;10^{3}$	3.29 $\times \;10^{3}$	3.24 $\times \;10^{3}$	3.26 $\times \;10^{3}$	3.31 $\times \;10^{3}$
	Std	2.19 $\times \;10^{1}$	2.24 $\times \;10^{1}$	1.52 $\times \;10^{1}$	2.36 $\times \;10^{1}$	3.13 $\times \;10^{1}$
	Rank	2	4	1	3	5
F29	Mean	3.55 $\times \;10^{3}$	3.84 $\times \;10^{3}$	3.60 $\times \;10^{3}$	3.62 $\times \;10^{3}$	3.87 $\times \;10^{3}$
	Std	7.83 $\times \;10^{1}$	2.04 $\times \;10^{2}$	1.05 $\times \;10^{2}$	1.10 $\times \;10^{2}$	1.82 $\times \;10^{2}$
	Rank	1	4	2	3	5
	Mean Rank	1.13	3.73	2.47	2.67	5.00
	Final Rank	1	4	2	3	5

**Table 7:** Comparison results on the three-bar truss design problem.

Algorithm	Optimized Result			Optimization Variable	
	Best	Mean	Std.	$x_{1}$	$x_{2}$
EMPSO	263.89584	263.89585	0.000009	0.78869	0.40821
AWPSO	263.89625	265.59212	1.459546	0.78942	0.40614
PECSO	263.89592	264.19864	0.460413	0.78848	0.40879
WSO	263.89584	263.89589	0.000039	0.78872	0.40813
WOA	263.89636	263.95316	0.056557	0.787839929	0.41062
PSO	263.89584	263.89585	0.000003	0.78868	0.40824

**Table 8:** Comparison results on the pressure vessel design problem.

Algorithm	Optimized Result			Optimization Variable			
	Best	Mean	Std.	$x_{1}$	$x_{2}$	$x_{3}$	$x_{4}$
EMPSO	6059.71434	6073.06615	15.529393	12.85781	7.14422	42.09845	176.63660
AWPSO	6319.46259	7826.71843	663.786354	13.06618	7.50000	40.71069	198.30049
PECSO	6059.71431	6355.17383	376.004535	12.81252	6.43752	42.09485	176.63661
WSO	6059.71473	6208.49219	106.840886	12.52765	6.97388	42.09845	176.63661
WOA	6129.15647	6728.76216	388.105378	14.44860	6.74448	45.00141	143.70370
PSO	6059.71434	6102.56744	75.657230	12.69574	7.04475	42.09845	176.63660

**Table 9:** Comparison results on the tension/compression spring design problem.

Algorithm	Optimized Result			Optimization Variable		
	Best	Mean	Std.	$x_{1}$	$x_{2}$	$x_{3}$
EMPSO	0.01267	0.01271	0.000020	0.05170	0.35702	11.27100
AWPSO	0.01389	0.01875	0.003225	0.05127	0.34308	13.40521
PECSO	0.01272	0.01321	0.000806	0.05179	0.35902	11.15565
WSO	0.01267	0.01290	0.000243	0.05173	0.35779	11.22652
WOA	0.01267	0.01296	0.000212	0.05172	0.35739	11.24984
PSO	0.01267	0.01271	0.000016	0.05143	0.35062	11.65546

**Table 10:** Statistical Comparison of EMPSO and BPSO for the OPP.

Problems	Algorithms	Optimal	Sub-Optimal	Avg.	Std.
IEEE 30-bus	EMPSO	10	12	10.50	0.629724
	BPSO	10	11	10.23	0.430183
	BBA	11	13	11.77	0.568321
IEEE 39-bus	EMPSO	13	18	15.77	1.16511
	BPSO	15	17	16.36	0.614948
	BBA	17	19	18.13	0.571346
IEEE 57-bus	EMPSO	20	25	22.67	1.39786
	BPSO	21	25	23.77	1.10433
	BBA	23	28	26.73	1.08066
IEEE 118-bus	EMPSO	44	60	52.53	3.32942
	BPSO	51	60	56.40	2.17509
	BBA	61	67	65.37	1.37674

**Table 11:** Optimal PMU placement obtained by EMPSO on different IEEE test systems.

Test System	Total Buses	PMUs Required	Optimal PMU Locations
IEEE 30-Bus	30	10	{2, 4, 6, 10, 11, 12, 15, 20, 25, 27}
IEEE 39-Bus	39	13	{2, 6, 9, 10, 11, 14, 17, 19, 20, 22, 23, 25, 29}
IEEE 57-Bus	57	20	{1, 4, 7, 10, 13, 20, 22, 24, 28, 30, 32, 35, 39, 41, 44, 47, 50, 53, 55, 56}
IEEE 118-Bus	118	44	{3, 5, 7, 8, 9, 12, 15, 17, 20, 23, 24, 25, 29, 35, 38, 40, 43, 47, 49, 50, 51, 52, 57, 59, 60, 64, 66, 68, 72, 73, 75, 76, 77, 78, 85, 86, 89, 92, 96, 100, 105, 107, 110, 115}

## Data Availability

The original contributions presented in this study are contained in this paper. Further inquiries can be directed to the corresponding author.

## Abbreviations

The following abbreviations are used in this manuscript:

APU	Adaptive Position Update
AWPSO	Adaptive Weighted Particle Swarm Optimizer
BBA	Binary Bat Algorithm
BPSO	Binary Particle Swarm Optimization
EEL	Elite Exemplar Learning
EMPSO	Exemplar Learning and Memory Retrieval-Based Particle Swarm Optimization
KLDE	Knowledge Learning Differential Evolution
MPSO	Modified Particle Swarm Optimization
OPP	Optimal Phasor Measurement Units Placement
PECSO	Performance-Enhanced Chicken Swarm Optimization
PMU	Phasor Measurement Units
PSO	Particle Swarm Optimization
SI	Swarm Intelligence
SMR	Superior Memory Recall
WOA	Whale Optimization Algorithm
WSO	White Shark Optimizer
